# A kiwifruit (*Actinidia deliciosa*) R2R3‐MYB transcription factor modulates chlorophyll and carotenoid accumulation

**DOI:** 10.1111/nph.15362

**Published:** 2018-08-01

**Authors:** Charles Ampomah‐Dwamena, Amali H. Thrimawithana, Supinya Dejnoprat, David Lewis, Richard V. Espley, Andrew C. Allan

**Affiliations:** ^1^ The New Zealand Institute for Plant & Food Research Limited (PFR) Private Bag 92 169 Auckland New Zealand; ^2^ The New Zealand Institute for Plant & Food Research Limited (PFR) Private Bag 11600 Palmerston North 4442 New Zealand; ^3^ School of Biological Sciences University of Auckland Private Bag 92019 Auckland New Zealand

**Keywords:** carotenoid, chlorophyll, kiwifruit, overexpression, transcription factor, transcriptomics

## Abstract

- MYB transcription factors (TFs) regulate diverse plant developmental processes and understanding their roles in controlling pigment accumulation in fruit is important for developing new cultivars. In this study, we characterised kiwifruit TF *MYB7*, which was found to activate the promoter of the kiwifruit *lycopene beta‐cyclase* (*AdLCY‐*β) gene that plays a key role in the carotenoid biosynthetic pathway.
- To determine the role of *MYB7*, we analysed gene expression and metabolite profiles in *Actinidia* fruit which show different pigment profiles. The impact of *MYB7* on metabolic biosynthetic pathways was then evaluated by overexpression in *Nicotiana benthamiana* followed by metabolite and gene expression analysis of the transformants.
- *MYB7* was expressed in fruit that accumulated carotenoid and Chl pigments with high transcript levels associated with both pigments. Constitutive over‐expression of *MYB7*, through transient or stable transformation of *N. benthamiana*, altered Chl and carotenoid pigment levels. *MYB7* overexpression was associated with transcriptional activation of certain key genes involved in carotenoid biosynthesis, Chl biosynthesis, and other processes such as chloroplast and thylakoid membrane organization.
- Our results suggest that *MYB7* plays a role in modulating carotenoid and Chl pigment accumulation in tissues through transcriptional activation of metabolic pathway genes.

## Introduction

**Figure 1:**
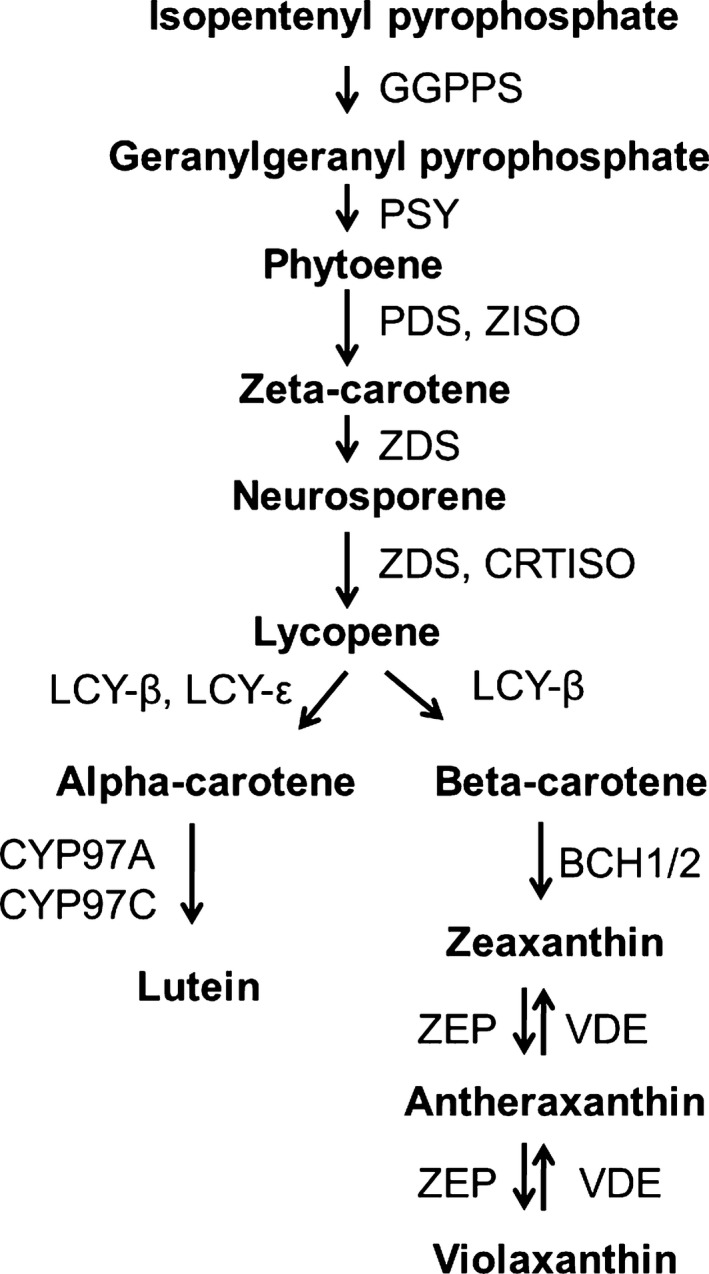
A schematic of the carotenoid biosynthetic pathway in plants. Enzymes involved in the various conversion steps are indicated with arrows. GGPPS, geranylgeranyl pyrophosphatase; PSY, phytoene synthase; PDS, phytoene desaturase; ZISO, zeta‐carotene isomerase; ZDS, zeta‐carotene desaturase; CRTISO, carotenoid isomerase; LCY‐β, lycopene beta‐cyclase; LCY‐ɛ, lycopene epsilon‐cyclase; BCH, beta‐carotene hydroxylase; CYP97A, cytochrome P450 beta‐ring carotenoid hydroxylase; CYP97C, cytochrome P450 epsilon‐ring carotenoid hydroxylase; ZEP, zeaxanthin epoxidase; VDE, violaxanthin de‐epoxidase.

The carotenoid biosynthetic pathway (Fig. 1) has been well characterised in various plant species and it is known to be controlled by factors such as channelling pathway flux, limiting enzyme steps or the availability of storage structures (Cunningham, 2002; Cazzonelli & Pogson, 2010; Yuan *et al*., 2015). The control of the pathway by either metabolic flux or a limiting biosynthetic step is related to enzyme activity, which is partly regulated by the modulation of gene expression. Transcriptional regulation of the carotenoid pathway has been implicated in various studies showing quantitative association between steady‐state transcript levels and pigment accumulation in tissues. In tomato, the accumulation of lycopene has been partly attributed to the downregulation of lycopene cyclase genes, while the expression of upstream genes such as phytoene synthase (*PSY*) increases carotenoid concentration (Fraser *et al*., 1994, 2007). *PSY* plays a significant role in controlling metabolic flux down the pathway because it is the first committed step in the carotenoid pathway (Rodriguez‐Villalon *et al*., 2009). This is essential as the carotenoid pathway shares the same substrate precursor, geranylgeranyl pyrophosphate, with other pathways such as the Chl, tocopherol and GA_3_ biosynthesis (Lu *et al*., 2013). In *Arabidopsis*, a single *PSY* is present and therefore the regulation of transcript abundance is essential to this step (Rodriguez‐Villalon *et al*., 2009; Cazzonelli & Pogson, 2010). In other species such as maize, rice, apple and loquat, some redundancy is provided by multiple *PSY* genes (Li *et al*., 2008; Welsch *et al*., 2008; Fu *et al*., 2014; Ampomah‐Dwamena *et al*., 2015). The tissue‐specific expression observed between different *PSY*s in these species suggests a tightly regulated transcription of this step. The PSY step is also post‐transcriptionally regulated by OR/OR‐like proteins affecting PSY protein levels and carotenoid content in Arabidopsis and sweet potato without affecting *PSY* transcript levels (Zhou *et al*., 2015; Park *et al*., 2016). Recently, it has been reported that the OR protein, with the golden SNP mutation, may increase carotenoid content by negatively regulating the downstream beta‐carotene hydroxylase activity in melon (Chayut *et al*., 2017).

Different kiwifruit cultivars and species display a wide range of fruit coloration due to the accumulation of pigments such as Chl, anthocyanin and carotenoids (Montefiori *et al*., 2005; Crowhurst *et al*., 2008). Developing new varieties is challenging due to the dioecism and extensive variation in ploidy levels present in the genus *Actinidia*. Understanding the genetic factors controlling carotenoid accumulation is valuable to plant breeding efforts to generate novel fruit phenotypes. The transcriptional control of carotenoid accumulation has been studied in kiwifruit with different fruit pigmentation, and the expression level of the *lycopene beta‐cyclase* (*LCY‐*β) gene was consistent with the accumulation of beta‐carotene and lutein as the dominant carotenoid compounds in fruit (Ampomah‐Dwamena *et al*., 2009). The coordinated transcriptional control of carotenoid metabolism in kiwifruit suggests that transcription factors (TFs) may play a role in regulation of this pathway. However, the mechanisms involved are not fully understood.

TFs regulate many developmental and physiological processes in plants via their ability to bind to promoters of target genes to control gene expression. A number of TFs have been implicated in the carotenoid pathway (Ye *et al*., 2015). MADS‐box genes *AGAMOUS‐like 1* and *FRUITFULL* have been found to regulate carotenoid accumulation during ripening in tomato (Vrebalov *et al*., 2009), and tomato ripening‐inhibitor (RIN), through its interaction with the *PSY1* promoter, has been shown to regulate fruit carotenoid concentrations (Martel *et al*., 2011). TFs from the AP2/ERF subgroup have been reported as playing significant roles in carotenoid accumulation (Welsch *et al*., 2007; Chung *et al*., 2010; Lee *et al*., 2012). In tomato, the NAC TFs SlNAC1 and SlNAC4 have been shown to modulate carotenoid accumulation during fruit ripening (Ma *et al*., 2014; Zhu *et al*., 2014).

Two recent publications have now described a role for MYB TFs in carotenoid regulation. Plant MYBs comprise a large superfamily and are implicated in diverse roles from flower and seed development, to hormone signalling, metabolite biosynthesis and tissue pigmentation as reviewed by Jin & Martin (1999). The first of these papers reveals an R2R3 MYB, Reduced Carotenoid Pigmentation 1 (RCP1), from *Erythranthe* (*Mimulus*) *lewisii* (Sagawa *et al*., 2016). This was identified through bulk segregant analysis of *E*. *lewisii* as a positive regulator of carotenoid levels in flowers. In a mutant of *E*. *lewisii* with reduced petal colour, it was shown that *RCP1* controls the entire carotenoid biosynthetic pathway. *RCP1* belongs to the MYB subgroup 21 (according to Stracke *et al*., 2001) which, to date, has no known role in plant pigment regulation. The second recent paper to describe a role for MYBs in carotenoid accumulation presents a very different mode of action. From studies using a *Citrus reticulata* stay‐green mutant, Green Ougan (MT), and the revertant, de‐greening wild‐type‐like Green Ougan (MT‐WT), the authors were able to identify an MYB TF, *CrMYB68*, which appeared to be negatively correlated with the activity of carotenoid pathway genes β*‐carotene hydroxylase 2* (*CrBCH2*) and 9*‐cis‐epoxycarotenoid dioxygenase 5* (*CrNCED5*) (Zhu *et al*., 2017). These two papers, while offering very different roles for MYBs, show that MYB TFs are involved in carotenoid regulation.

In fruit, carotenoid accumulation coincides with the fruit ripening process and the associated complex regulatory environment makes ascertaining the direct role of such TFs challenging. TFs involved in plant development are commonly identified through analysis of their causative phenotypes. However, identification of TFs controlling carotenoid biosynthetic pathway from this approach has been limited, possibly due to lethality of such mutations in plants. Therefore, promoter traps, deep sequencing methods or the binding of TF candidates to gene promoters have become important in understanding the regulation of the carotenoid pathway (Welsch *et al*., 2007; Vrebalov *et al*., 2009; Martel *et al*., 2011; Ye *et al*., 2015; Bond *et al*., 2016). Here we have used the *AdLCY‐*β gene promoter of kiwifruit in an *in planta* screen assay to identify a kiwifruit R2R3 MYB (*AdMYB7*) that binds to and activates the expression of *LCY‐*β. We characterised the *MYB7* gene in kiwifruit and analysed its overexpression in model plants. The results suggest that *MYB7* has a role in both Chl and carotenoid accumulation.

## Materials and Methods

### Plant material and growth conditions

Kiwifruit cultivars were selected from PFR orchards in Riwaka, New Zealand. Fruit from *Actinidia macrosperma* C.F. Liang and *Actinidia arguta* (Siebold & Zucc.) Planch. ex Miq. were picked at various stages of development from the vines. Fruit at mature green stage were stored at room temperature and sampled during ripening as described previously (Ampomah‐Dwamena *et al*., 2009). Plants for leaf infiltration with *Agrobacterium tumefaciens* were grown as described previously (Hellens *et al*., 2005). Transgenic *Nicotiana benthamiana* Domin plants were generated from leaf discs after inoculation with *Agrobacterium*‐carrying constructs as described previously for *Nicotiana tabacum* (Kalantidis *et al*., 2002). Transgenic plants were transferred to soil to grow under containment glasshouse conditions. Seeds from T1 plants were sterilised and grown on kanamycin (50 mg l^−1^) selection plates to produce T2 plants used for analysis.

### Plant transformation vectors

Kiwifruit MYB TF genes were identified from expressed sequence tag libraries (Crowhurst *et al*., 2008). cDNAs were amplified and cloned into pHEX2 vector using the Gateway cloning strategy as previously described (Hellens *et al*., 2005). Kiwifruit *lycopene beta cyclase* promoter fragment including 5′‐untranslated region (1.1 kb) was amplified from kiwifruit genomic DNA using primers LCYBpro F (ACTGCTGTAGCCTGTACTGTCA) and LCYBpro R (CCTGAGAAGAGTTCCCATAAGA). For promoter deletion constructs, primers Pro500 F (CAGCACCATGCTTTATTTGA) and Pro200 F (TGGTGAATCTCGTGCAGCATTC) were used in combination with LCYBpro R (CCTGAGAAGAGTTCCCATAAGA) to amplify 500 bp and 200 bp promoter fragments, respectively. PCR fragments were initially cloned into pGEM‐T Easy vector (Promega) for sequence confirmation and cloned into the *Not*I site in pGreen II 0800‐LUC upstream of the Luciferase reporter gene (Hellens *et al*., 2005).

### Transient assay of promoter activation

*AdLCY‐*β promoter sequences were analysed with plantpan 2.0 software (Chang *et al*., 2008) to identify binding motifs. Transient assays were performed as previously described (Ampomah‐Dwamena *et al*., 2009). *Agrobacterium tumefaciens* strain GV3101 carrying the pSOUP helper plasmid with the *LCY‐*β promoter construct or MYB construct were resuspended in infiltration buffer (10 mM MgCl_2_, 0.5 μM acetosyringone) and infiltrated into the abaxial side of *N. benthamiana* leaves. The plants were left to grow for 2 d before 2 mm leaf discs were taken from infiltrated leaves and assayed with a Victor ×3 Multi‐label Microplate Reader (Perkin Elmer, Waltham, MA, USA). Luminescence from Firefly Luciferase (LUC) expression driven by the *LCY‐*β promoter relative to Renilla (REN) luciferase signal activity under control of the *Cauliflower mosaic virus* 35S promoter was measured and expressed as a ratio (LUC/REN).

### Phylogenetic analysis and tree construction

A phylogenetic tree was constructed using Geneious 8.1.2 (Kearse *et al*., 2012) and mega7 (Kumar *et al*., 2016) software with *Arabidopsis* and kiwifruit MYB sequences retrieved from the GenBank database (Supporting Information Table S1). Genetic distances were calculated using the Jukes–Cantor distance matrix and evolutionary relationships were inferred using the maximum likelihood method with 1000 bootstrap resampling.

### Expression and purification of recombinant protein

*AdMYB7* cDNA encoding the open reading frame was amplified with MYB281F (ATGGAAGTTGGAGGCAGAGT) and MYB281R (CATGTTGAATTGTTGTTGTAG) primers, modified to contain the *Nde*I restriction site. The amplified fragment was cloned into the *Nde*I site of pET30b vector to generate a C‐terminal histidine‐fused protein. The cloned vector was sequenced, confirmed and transformed into BL21‐CodonPlus‐RIL competent cells (Stratagene, San Diego, CA, USA). Recombinant protein purification followed an earlier method developed by Green *et al*. (2007). A 500 ml culture containing ZYM‐5052 auto‐induction media (Studier, 2005) was grown at 16°C, 300 rpm for 3 d to obtain an OD_600_ of 12–15. Cells were harvested by centrifugation and frozen at −80°C overnight. The cells were thawed at 4°C in 50 ml 1× His Trap Binding buffer (50 mM NaH_2_PO_4_, 300 mM NaCl, pH 8) plus an EDTA‐free protease inhibitor cocktail tablet (Roche, Penzberg, Germany) and disrupted by passing twice through an EmulsiFlex‐C15 high‐pressure homogeniser (Avestin, Mannheim, Germany) with a pressure setting of 15 000 psi. The sample was centrifuged 32 000 ***g*** for 30 min (4°C) to remove cell debris and filtered through a 0.45 μm filter (Merck Millipore). The supernatant was loaded onto a precharged and equilibrated 5 ml His Trap column (GE Healthcare, Piscataway, NJ, USA) and washed with 30 ml 1× His Trap binding buffer (50 mM NaH_2_PO_4_, 300 mM NaCl, 15–35 mM imidazole, pH 8). Bound proteins were eluted at 2 ml min^−1^ using 0–500 mM imidazole gradient, pH 8, and analysed by SDS‐PAGE.

### Protein mobility shift assay

Radioactively labelled DNA probe from a *AdLCY‐*β promoter fragment (*c*. 200 bp) was incubated with recombinant His‐tagged kiwifruit *Ad*MYB7 protein at room temperature for 30 min, in a binding buffer (10 mM Tris, pH 7.5, 1 mM EDTA, 100 mM KCl, 0.1 mM dithiothreitol, 5% glycerol and 0.01 mg ml^−1^ BSA) with poly dI.dC (1 μg μl^−1^) as nonspecific competitor. For specific competition, unlabelled DNA probe was added to the reaction. Samples were then loaded onto 4% polyacrylamide gel in TBE buffer (pH 9.2) and run at 180 V for 40 min. The electrophoresed gel was detected using an X‐ray film (Kodak).

### HPLC pigment analysis

High‐performance liquid chromatography (HPLC) analysis was performed on a Dionex Ultimate 3000 solvent delivery system (Thermo Scientific, Waltham, MA, USA) fitted with a YMC RP C30 column (5 μm, 250 × 4.6 mm), coupled to a 20 × 4.6 C30 guard column (YMC Inc., NC, USA) and a Dionex 3000 PDA detector as previously reported (Ampomah‐Dwamena *et al*., 2015). Phytoene was monitored at 280 nm and coloured carotenoids and Chl*b* were detected at 450 nm, while Chl*a* and its derivatives were monitored at 400 nm. Carotenoid concentrations were determined as β‐carotene equivalents per gram dry weight (DW) of tissue. All trans‐β‐carotene, lutein and Chl*a* standards were purchased from Sigma Chemicals. Other carotenoids were putatively identified by comparison with reported retention times and spectral data. Total carotenoid and Chl content of the fruit tissue was also estimated using methods as previously described (Wellburn, 1994).

### RNA extraction and cDNA synthesis

Total RNA was extracted by tissue homogenisation in CTAB buffer using a modified method from one previously described (Chang *et al*., 1993). Complementary DNA (cDNA) was synthesised from total RNA (*c*. 1 μg) using a Quantitec reverse transcription kit (Qiagen) following the manufacturer's protocol. RNA samples were treated with the genomic DNA wipeout buffer followed by reverse transcription reaction. Reaction components included RT primer mix, Quantiscript reverse transcription buffer and Quantiscript reverse transcriptase. The reactions were incubated at 42°C for 30 min followed by 95°C for 3 min to inactivate enzyme.

### Quantitative real‐time PCR analysis

Primers used (Table S2) were designed using Primer3 software (Rozen & Skaletsky, 2000) to a stringent set of criteria. Real‐time quantitative PCR (RT‐qPCR) was performed under conditions described previously (Lin‐Wang *et al*., 2010). First‐strand cDNA products were diluted 1 : 25 and used as templates for the PCR. PCR analysis was performed using the LightCycler 1.5 system and the SYBR Green master mix (Roche), following the manufacturer's protocol. Each reaction sample was analysed from biological replicates, with a negative control using water as template. PCR conditions were as follows: pre‐incubation at 95°C for 5 min followed by 40 cycles each consisting of 10 s at 95°C, 10 s at 60°C and 20 s at 72°C. Amplification was followed by a melting curve analysis with continuous fluorescence measurement during the 65–95°C melt (Nieuwenhuizen *et al*., 2013). The relative expression was calculated using LightCycler software v.4 and the expression of each gene was normalised to a reference gene with stable expression in these tissues.

### Transcriptome analysis by RNASeq

*Agrobacterium*‐infiltrated *N. benthamiana* leaves were harvested after 24 h (T1) or 72 h (T2) and frozen in liquid nitrogen. Total RNA was extracted as described above and sequencing libraries were prepared using the TruSeq mRNA library preparation kit (Illumina) and sequenced on a HiSeq2000 (Illumina) using 2 × 100 bp paired‐end sequencing. Each treatment had three biological replicates, with each replicate being a pool of leaves from individual plants. The libraries were multiplexed and run on three lanes generating 48–54 million reads per sample. Resulting reads were then quality trimmed where fastq‐mcf (ea‐utils.1.1.2‐806) was used for adapter removal with a quality threshold cut‐off of 20, followed by the use of an in‐house perl script to trim 15 bases of the 5′‐end and remove any reads with N's or mononucleotides. Thereafter, reads were aligned to the Solgenomics *N*. *benthamiana* genome v.1.0.1 (https://solgenomics.net/organism/Nicotiana_benthamiana/genome) using Bowtie2 (v.2.2.5). The number of reads aligning to the annotated genes was counted using HTSeq (v.0.6.1p1). Differentially expressed genes were identified using DESeq2 analysis with a cut‐off probability of *P *<* *0.05. Gene ontology (GO) enrichment analysis was performed using david (Huang *et al*., 2009) with TAIR10 annotation. The GOplot R package was used to visualise the GO enrichment analysis (Walter *et al*., 2015).

## Results

### *AdMYB7* activates the kiwifruit *LCY‐*β promoter

**Figure 2:**
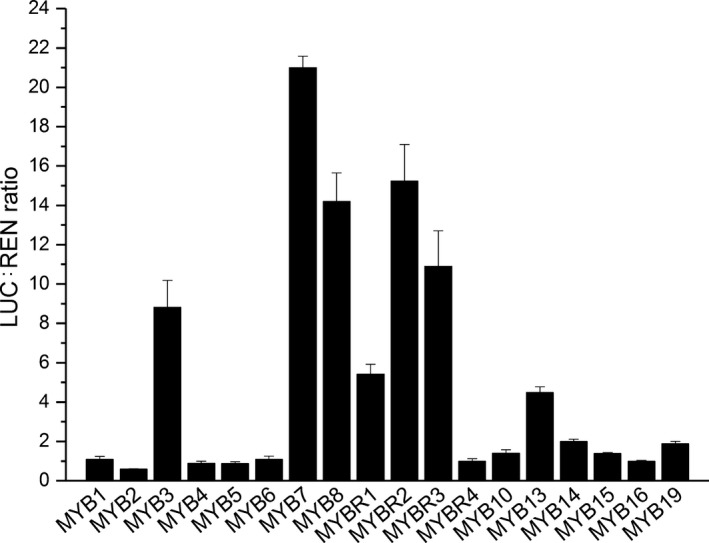
Transient activation of *AdLCY‐*β promoter by kiwifruit MYB transcription factors. Ratio of luminescence (LUC) signals measured from *Nicotiana benthamiana* leaves coinfiltrated with *Agrobacterium* constructs of MYB genes and a vector with firefly luciferase under the control of *LCY*‐β promoter and Renilla luciferase (REN) under the control of CaMV 35S promoter. Luminescence signals were normalised to the basal promoter activity. Bars represent means ± SE of four biological replicates.

**Figure 3:**
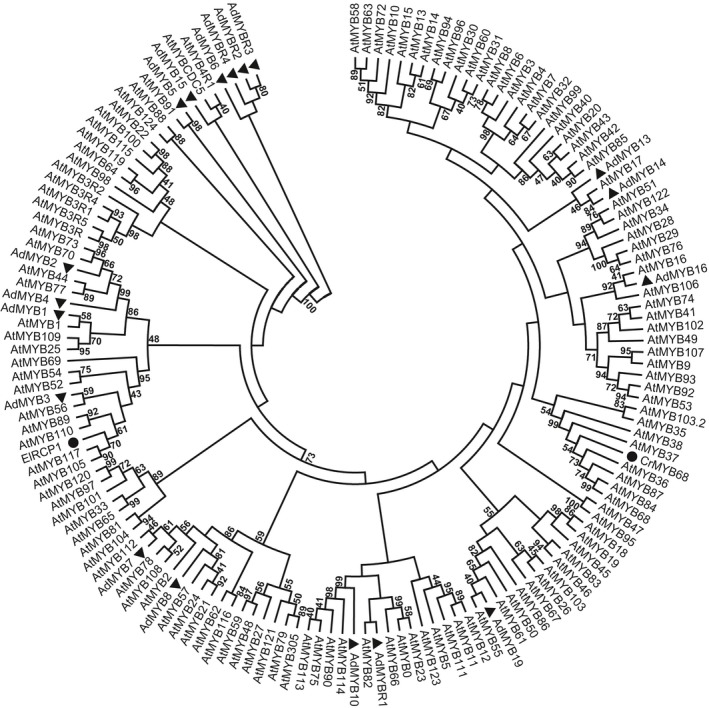
Phylogenetic tree of MYB transcription factors showing the relationship between kiwifruit MYB sequences in this study, MYBs implicated in carotenoid regulation (ElRCP1 and CrMYB68) and sequences retrieved from *Arabidopsis* databases. Protein sequences were aligned using ClustalW and evolutionary relationships were determined using maximum‐likelihood analysis with 1000 bootstrap replicates. The kiwifruit sequences are indicated by triangles, ElRCP1 and CrMYB68 by circles. GenBank accession numbers are provided in Supporting Information Table S1.

To identify transcriptional regulators of *AdLCY‐*β, a 1.1 kb promoter fragment was amplified by PCR from *A. deliciosa* genomic DNA and the sequence was analysed for the presence of gene regulatory motifs using plantPAN 2.0 (Chang *et al*., 2008). Motifs identified included those implicated in abscisic acid, light and stress response as well as MYB binding sites (Fig. S1). Due to the importance of MYBs in controlling a variety of plant‐specific processes (Ambawat *et al*., 2013), we tested a number of MYBs for their activation of the *AdLCY‐*β promoter. The promoter fragment was cloned upstream of the *Luciferase* gene in the pGreen 0800 LUC vector (Hellens *et al*., 2005) and co‐infiltrated with *Agrobacterium* transformation vectors carrying kiwifruit MYB TFs into young leaves of *N. benthamiana*. Most of the MYB constructs activated the promoter to some extent, measured by increases in the ratio of luminescence signals between the *AdLCY‐*β and *Cauliflower mosaic virus* 35S promoters (Fig. 2). This screen relies on the assumption that high translation of each construct results in high protein concentration and structure, which can be partially addressed by replication of each assay and support of other techniques. *Ad*MYB7 (best blast match to AtMYB112), MYBR2 and MYBR3 (both best blast matches to AtMYB88), MYB8 (match, AtMYB21), and MYB3 (match, AtMYB89) showed strong activation of the promoter, suggesting binding specificity of certain MYBs to the promoter. Among these, *Ad*MYB7 was selected for further analysis based on its high promoter activation and gene expression pattern in fruit. *Ad*MYB7 belongs to the phylogenetic clade including AtMYB112, AtMYB108 and AtMYB78 (Fig. 3; Stracke *et al*., 2001) and shows 53%, 49% and 48% amino acid sequence identity, respectively, to these *Arabidopsis* proteins. The *Ad*MYB7 sequence was analysed with wolf psort (Horton *et al*., 2007), an advanced protein subcellular localisation prediction tool, which predicted *Ad*MYB7 as a nuclear localised protein, consistent with R2R3 MYB proteins.

### *AdMYB7* binds the *AdLCY‐*β promoter

**Figure 4:**
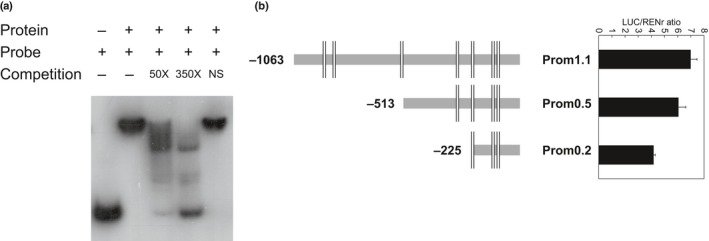
*Ad* MYB7 binds and enhances the activity of the *AdLCY‐*β promoter. (a) EMSA analysis using a ^32^P‐labelled 200 bp promoter fragment showed that MYB7 recombinant protein binds to the *LCY‐*β promoter fragment. The migration of the labelled probe only is shown in lane 1. Complexes formed between labelled probe and MYB7 protein (lane 2), with competition from 50‐fold and 350‐fold increase of unlabelled probe, are shown in lanes 3 and 4, and with nonspecific competitor DNA (NS) in lane 5. (b) Constructs of *AdLCY‐*β promoter deletion fragments (showing MYB binding sites predicted by Plant PAN 2.0) infiltrated with *Ad* MYB7 construct as described above. Data are means ± SE of four biological replicates.

To further confirm the interaction seen in the luciferase reported assays, the physical binding of MYB7 recombinant protein to the *AdLCY‐*β promoter fragment was tested using an electrophoretic mobility shift assay (EMSA). The nucleotide coding region of *AdMYB7* was amplified from fruit cDNA, cloned into the *Nde*I site of pET30 vector (Novagen) and expressed as a 38.7 kDa recombinant protein (339 amino acids, pI = 6.04) with a C‐terminal His‐tag. A specific DNA–protein complex was formed between *Ad*MYB7 recombinant protein and the promoter fragment, and its migration was slower when compared with the labelled probe only (Fig. 4a). The formation of this complex was reduced when an increasing amount of the unlabelled promoter fragment, as a competitor, was added. By contrast, no competition was observed when an amplified fragment from kiwifruit actin cDNA was used as competitor, suggesting specificity in DNA–protein complex formation. Analysis of the promoter deletion constructs of the *AdLCY‐*β promoter by the dual luciferase assay showed that activation of the 0.5 kb fragment by *Ad*MYB7 was reduced compared with the longer 1.1 kb fragment while the 0.2 kb fragment was least activated (Fig. 4b).

### *MYB7* gene expression in kiwifruit

**Figure 5:**
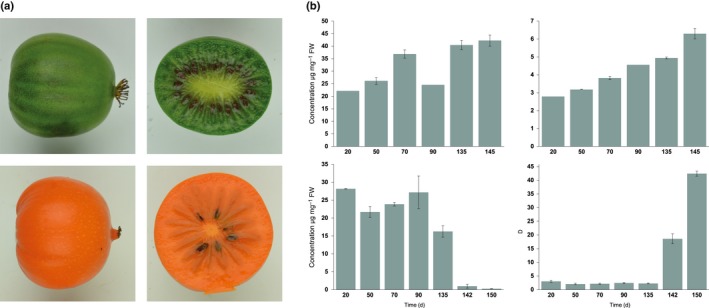
(a) Ripe fruit of *Actinidia arguta* (green) and *Actinidia macrosperma* (orange). (b) Total Chl (left) and total carotenoid concentration (right) in fruit of *A*. *arguta* (top panel) and *A*. *macrosperma* (bottom panel) during fruit development. Error bars indicate ± SE from three biological replicates.

To understand the role of *MYB7* in fruit, we examined expression levels in two kiwifruit species, *A. arguta*, which has green fruit throughout its development, and *A. macrosperma*, which turns bright orange when ripe (Fig. 5a). In *A*. *arguta* fruit, total Chl concentration increased gradually from 20 d after full bloom (DAFB) to 145 DAFB, although a decrease in concentration was observed at 90 DAFB. Total carotenoid concentration remained relatively low throughout fruit development (Fig. 5b). In *A*. *macrosperma* fruit, Chl concentration was high at the early fruit stages and the level remained stable until fruit reached 90 DAFB. Chl concentration then decreased sharply until it was barely detectable in the 150 DAFB ripe fruit. Total carotenoid concentration in this fruit, by contrast, was lower in the young fruit until after the 135 DAFB stage, when carotenoid concentration increased significantly in the ripening fruit at 142 and 150 DAFB. The dominant carotenoid compounds in both *A*. *arguta* and *A*. *macrosperma* were lutein and beta‐carotene, while zeaxanthin accumulated to higher concentration in *A. arguta* than in *A*. *macrosperma* fruit. Alpha‐carotene concentration were fairly stable throughout fruit development of *A*. *arguta*, but increased 10‐fold from 90 DAFB to the ripe fruit stages in *A*. *macrosperma* (Fig. S2).

**Figure 6:**
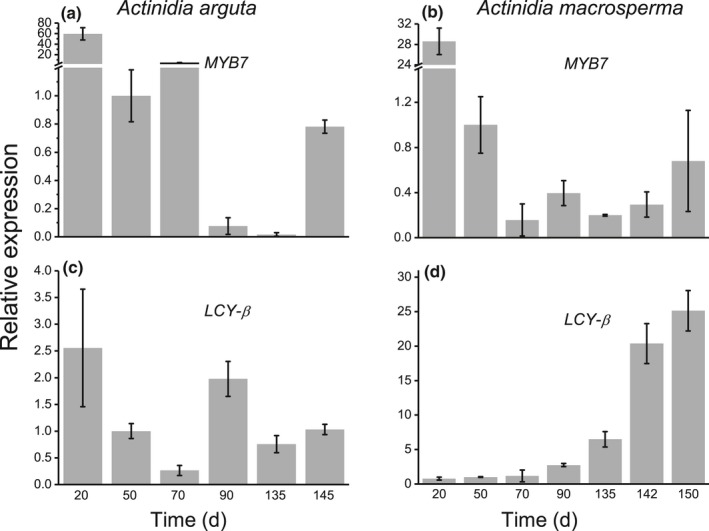
Gene expression profile of *MYB7* and *lycopene beta‐cyclase* (*LCY‐*β) in kiwifruit genotypes during fruit development. Relative gene expression of *MYB7* and *LCY‐*β in fruit of *Actinidia arguta* (a, c) and *Actinidia macrosperma* (b, d). Data were analysed using target : reference ratios (measured with Lightcycler 480 software) using actin as reference gene and presented as means ± SE from three biological replicates.

*MYB7* transcript level in *A*. *arguta* was very high at 20 DAFB, reduced at 50 DAFB and then an increase in expression was observed at 70 DAFB. Expression was reduced after this fruit stage until 145 DAFB (ripe fruit stage) when a final increase in expression was seen (Fig. 6a). A similar pattern for *MYB7* was seen in *A. macrosperma* fruit, with very high expression at 20 DAFB, decreasing from 50 DAFB until 142 DAFB (mature green), and increasing again at 150 DAFB (ripe fruit) (Fig. 6b). We then compared expression of the *LCY‐*β gene in these fruit. Its expression pattern in *A*. *arguta* fruit showed that the transcript level was high at 20 DAFB, was significantly reduced at 50 and 70 DAFB, and showed an increase at 90 DAFB. Expression was then reduced after this stage (Fig. 6c). By contrast, *AmLCY‐*β transcript levels in *A. macrosperma* fruit increased consistently from 20 to 150 DAFB (Fig. 6d).

We also examined the gene expression in fruit of the other MYB TF candidates, which activated the *AdLCY*‐β promoter (Fig. S3). Transcript levels of *MYB8* and *MYBR2* were not detected in fruit. The transcript levels of *MYB3*,* MYB13* and *MYBR3* were high at 20 DAFB in both *A. arguta* and *A*. *macrosperma* and then were reduced as the fruit developed to maturity. By contrast, *MYBR1* expression in *A. arguta* was undetectable in the 20 DAFB fruit but increased in 50 and 70 DAFB fruit, after which expression decreased. In *A. macrosperma*,* MYBR1* expression was low at the 20 and 50 DAFB fruit stages and transcript levels then increased in the 70, 90 and 135 DAFB fruit stages before decreasing in the later stages. Transcript levels of key carotenoid pathway genes were also assessed in the fruit of both kiwifruit species. The expression pattern for all the genes in *A*. *arguta* showed high transcript levels of the genes at 20 DAFB but reduced thereafter, except for the *BCH* gene for which expression appeared to increase at 90 DAFB (Fig. S4A). In *A*. *macrosperma*, the expression pattern for *PSY*,* PDS* and *ZDS* was similar with high transcript levels at 20 DAFB but reduced at 50 and 70 DAFB before increasing again from 90 to 150 DAFB. By contrast, *CRTISO*,* LCY‐*ɛ and *BCH* gene expression in *A*. *macrosperma* fruit showed high transcript levels at 20 DAFB before reducing thereafter to 150 DAFB (Fig. S4B).

### Overexpression of *AdMYB7* alters pigment accumulation in *Nicotiana benthamiana*

To analyse the effects of *AdMYB7* expression on carotenoid levels, *N*. *benthamiana* leaves were infiltrated with *Agrobacterium* carrying the *AdMYB7* cDNA cloned under the control of the *Cauliflower mosaic virus* 35S promoter (35S:*MYB7*). The leaves were left to grow on the plant for 5 d and changes in leaf colour and pigment profiles were analysed. There was a gradual yellowing of the *AdMYB7* infiltrated leaf patch, while the part of the leaf that was infiltrated with empty vector remained green (Fig. S5A). HPLC analysis of the leaves infiltrated with the *MYB7* construct showed a more than two‐fold increase in total carotenoid concentration (Fig. S5B).

**Figure 7:**
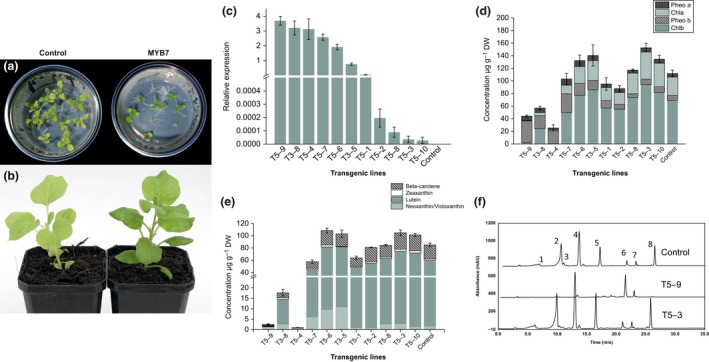
*Nicotiana benthamiana* plants expressing *AdMYB7* show changes in Chl and carotenoid profiles. *MYB7* transgenic plants displayed darker green leaves in tissue culture (a) and in soil (b). (c) Transcript levels of *MYB7* in the transgenic *N. benthamiana* plants measured by RT‐qPCR (arranged in decreasing order). Bars represent means and SE from four biological replicates. (d, e) Graphs showing concentrations of Chl pigments, pheophytin (Pheo) a, Chl*a*, Pheo b, Chl*b* (d) and carotenoid pigments (e) in transgenic *N*. *benthamiana* plants measured by HPLC from leaf tissue. Bars represent means and standard errors from three biological replicates. (f) HPLC pigment profiles showing pigments detected in 50 mg of leaves of control (top), T5‐9 (middle) and T5‐3 (bottom) transgenic plants. Labelled peaks: 1, violaxanthin; 2, lutein; 3, zeaxanthin; 4, Chl*b*; 5, Chl*a*; 6, pheophytin a; 7, pheophytin b; 8, beta‐carotene.

To further analyse *AdMYB7* function, we generated transgenic *N*. *benthamiana* plants using *Agrobacterium* transformation of leaf discs with a 35S: *AdMYB7* construct. Five independently transformed T1 plants were grown in soil under glasshouse conditions to obtain seeds. T2 plants from two of these transgenic lines were selected in tissue culture, after which they were grown in the glasshouse under long‐day conditions alongside empty vector control plants (Fig. 7a,b). The T2 transgenic plants showed varying levels of *AdMYB7* gene expression as measured by RT‐qPCR and could be grouped into high (T5‐9, T3‐8, T5‐4, T5‐7, T5‐6, T3‐5, T5‐1) and low (T5‐2, T5‐8, T5‐3, T5‐10) expression plants (Fig. 7c). In contrast to the transiently infiltrated *N*. *benthamiana* plants, the *AdMYB7* stable transgenic plants displayed darker green leaves compared with the control plants, which suggested increased accumulation of Chl in these tissues (Fig. 7a,b).

**Figure 8:**
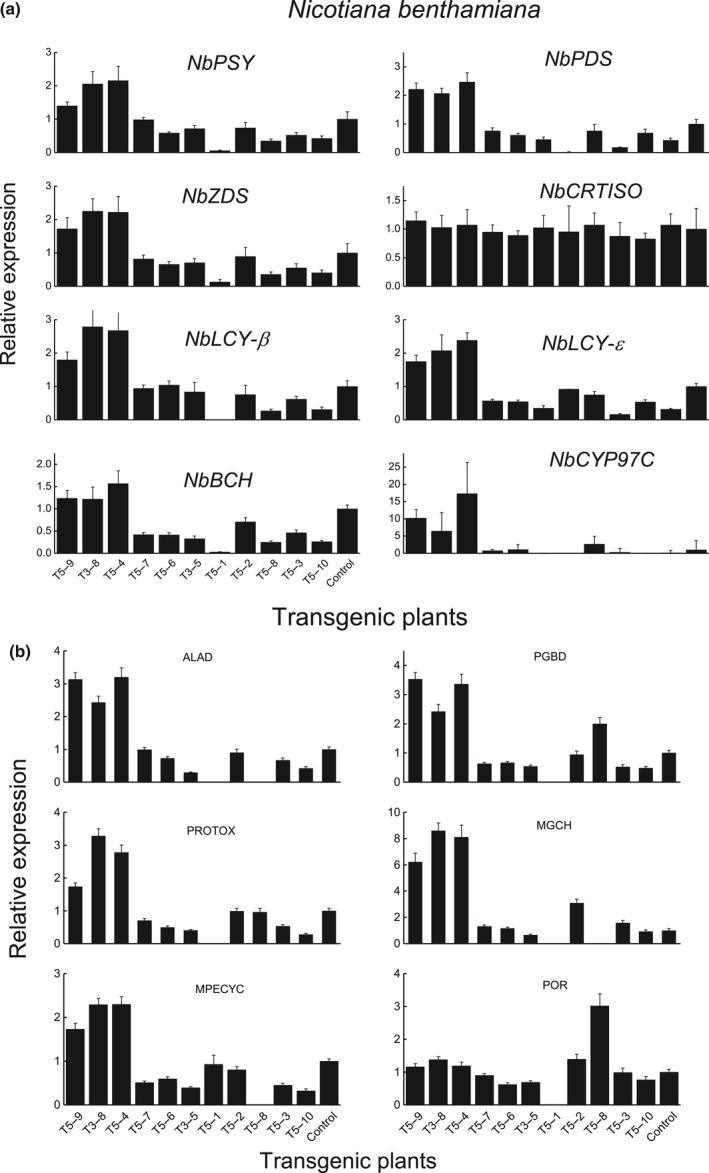
Relative expression of carotenoid (a) and Chl (b) biosynthetic pathway genes in transgenic MYB7 *Nicotiana benthamiana* plants. Values are means ± SE from three biological replicates. *PSY*, phytoene synthase; *PDS*, phytoene desaturase; *ZDS*, zeta‐carotene desaturase; *CRTISO*, carotene isomerase; *LCY*‐β, lycopene beta‐cyclase; *LCY*‐ɛ, lycopene epsilon‐cyclase; *BCH*, beta‐carotene hydroxylase; *NbCYP97C*, cytochrome P450 epsilon‐ring carotenoid hydroxylase; ALAD, delta‐aminolevulinic acid dehydratase; PGBD, porphobilinogen deaminase; PROTOX, protoporphyrinogen oxidase; MGCH, magnesium chelatase; MPECYC, magnesium protoporphyrin ester cyclase; POR, protochlorophyllide oxidoreductase.

Chlorophyll and carotenoid concentrations in the leaves were measured by HPLC to ascertain the effect of *AdMYB7* on pigment accumulation. In the seven high *MYB7* expression plants, total Chl concentration was reduced in four plants (T5‐9, 3‐8, 5‐4, 5‐1), was increased in two (T5‐6 and T3‐5), with one (T5‐7) showing a similar concentration to control plants (Fig. 7d). Pheophytin a/b compound concentrations, as a proportion of total Chl, were higher in these four plants than in the control or other transgenic plants, although artefactual conversion of Chl compounds can be caused by the extraction process (Hu *et al*., 2013). By contrast, the plants with lowest *MYB7* expression showed somewhat increased Chl concentrations compared with the control. The main carotenoid compounds detected in the transgenic plants were lutein and beta‐carotene. The pattern of total carotenoid accumulation observed between the transgenic lines was similar to the total Chl accumulation described previously. Five high *MYB7* expression plants (T5‐9, 3‐8, 5‐4, 5‐7 and 5‐1) showed reduced carotenoid concentrations, while T5‐6, T3‐5, T5‐3 and T5‐10 showed increased concentrations compared with the control. These results suggest that heterologous overexpression of *MYB7* in transgenic *N*. *benthamiana* plants can increase Chl and carotenoid concentrations, when expression of the transgene is at a moderate level. However, transgene expression above a certain threshold significantly reduced concentrations of these pigments. In addition to the changes in pigment levels, we observed stunted plant growth in the lines with high *MYB7* expression. T3‐8, T5‐4 and T5‐9, for instance, reached only a height of *c*. 10 cm compared with the control plants that grew to an average height of 24 cm. There was a negative correlation (*r *=* *−0.89, *P *<* *0.05) between transgene expression level and plant height, suggesting that *MYB7* overexpression affected plant growth. We examined the relative expression of carotenoid and Chl biosynthetic pathway genes in the transgenic *N. benthamiana* plants and found carotenoid genes such as *NbPSY*,* NbPDS*,* NbZDS*,* NbLCY‐*β and *NbLCY‐*ɛ were upregulated in the high *AdMYB7*‐expressing plants (Fig. 8a). A similar expression pattern was also observed with the Chl pathway genes such as delta‐aminolevulinic acid dehydratase, magnesium protoporphyrin ester cyclase, porphobilinogen deaminase and magnesium chelatase showing increased expression in these *AdMYB7* transgenic plants (Fig. 8b).

### Global transcriptional changes induced by *AdMYB7* in *Nicotiana benthamiana*

To examine the mechanism involved in *AdMYB7* regulation of pigment accumulation, we analysed the transcriptomes of *N*. *benthamiana* leaves agro‐infiltrated with a T‐DNA construct of *MYB7* compared with those carrying the empty vector (EV) or mock infiltrated with buffer. Transient expression of genes using *Agrobacterium* infiltration into *N*. *benthamiana* as a heterologous host has been used recently to validate gene function or determine protein localisation (Leckie & Stewart, 2011; Pillay *et al*., 2016). In this study, infiltrated leaves were left for either 24 h (T1) or 72 h (T2) on the plant before tissue was isolated for RNA extraction and analysis by transcriptome sequencing. By using RNAseq of transiently transfected *35S:MYB7* leaf patches*,* lists of differentially expressed tobacco genes resulting from the primary response to this TF are more likely to be revealed, rather than 4 or 5 wk of growth of a plant that has been affected by stable transformation.

The transcriptome of leaves agro‐infiltrated with empty vector or mock treatments (buffer only) were first compared. A significant number of genes were differentially expressed, suggesting a background response to the *Agrobacterium* with empty T‐DNA vector. At T1, 2659 and 2405 differentially expressed genes (DEGs) were found to be upregulated and downregulated, respectively, in empty vector vs buffer only, and at T2, 704 genes were upregulated and 35 genes downregulated (*P *<* *0.05). There were 549 upregulated and 12 genes downregulated common to both time points. With a log_2_ fold cut‐off (± 2) there were 414 DEGs responsive to *Agrobacterium* infection (384 at T1, 108 at T2 and 78 at both time points) (Table S3). The upregulated genes included those involved in defence response and disease resistance, such as receptor kinases, systemic resistance proteins, leucine‐rich repeat family proteins and WRKY TFs, indicating the plant's response to a perceived pathogen attack (Bond *et al*., 2016). The downregulated genes included some chloroplast‐ and photosynthesis‐related proteins.

**Figure 9:**
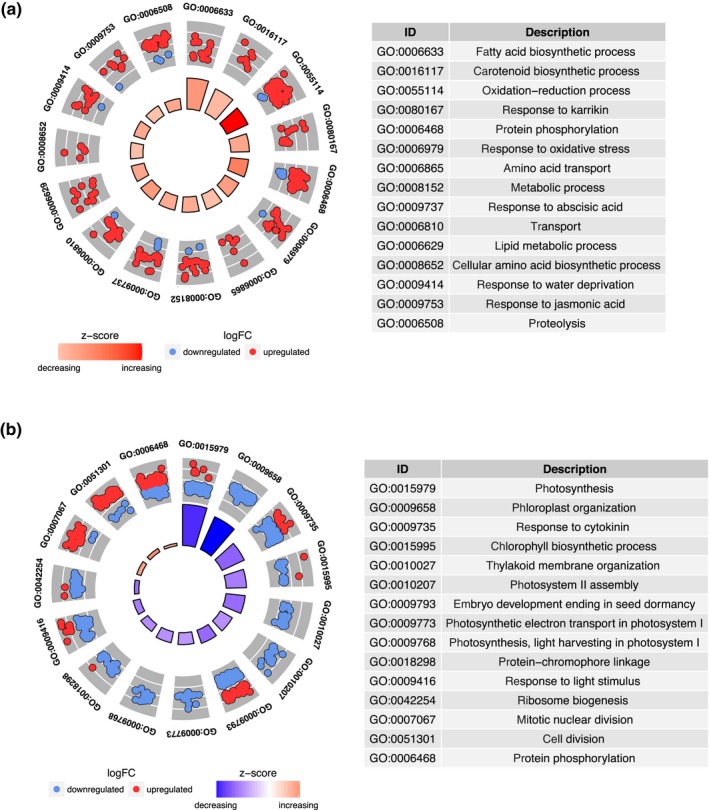
A GO plot of differentially expressed genes (*P *<* *0.05) of *AdMYB7*‐treated leaves in the biological process category at T1 (a) and T2 (b). The outer wheel shows a scatter plot of log fold change (FC) for each gene under the gene ontology (GO) terms. Red dots indicate upregulated genes and blue dots show downregulated genes; the inner wheel shows the *z*‐score, a measure of up‐ or downregulation, of each identified process.

The DEGs that were induced or repressed by transient expression of *MYB7* compared with EV were then analysed. There were 874 upregulated and 92 downregulated genes in *MYB7*‐treated leaves at T1 (24 h post‐infiltration). By contrast, a significantly higher number (>5‐fold) of differentially expressed genes were present at T2 (72 h): 2160 upregulated and 3042 downregulated genes, with 289 DEGs common to both time points (*P *<* *0.05). With a log_2_ fold cut‐off (± 2), there were 290 DEGs due to *AdMYB7* expression (Table S4). Some of the DEGs could be due to indirect response to *AdMYB7* expression and should be interpreted with caution. However, DEGs at T1 and those common to both time points are probably targets of *AdMYB7*. *Arabidopsis* homologues of the *N*. *benthamiana* DEGs were generated and analysed at *P *<* *0.05, for GO enrichment using the DAVID tool (Fig. 9; Huang *et al*., 2009). GO annotation analysis of both T1 and T2 DEGs was separated into the three main categories: biological process (BP), cellular component (CC) and molecular function (MF). For BP, the T1 DEGs were enriched for fatty acid biosynthesis (GO:0006633), carotenoid biosynthesis (GO:0016117) as well as oxidation‐reduction processes (GO:0055114), all with positive z‐scores that indicate these pathways were upregulated (Fig. 9a). By contrast, T2 DEGs were enriched for photosynthesis (GO:0015979), chloroplast organisation (GO:0009658), response to cytokinin (GO:0009735), Chl biosynthetic process (GO:0015995) and thylakoid membrane organisation (GO:0010027). However, these GO terms had negative z‐scores, indicating downregulation of these biological processes (Fig. 9b).

For CC annotations, T1 DEGs annotated as membrane‐associated genes (GO:0005886, GO:0016020, GO:0016021) made up the largest proportion and most of them were associated with the plasma membrane (GO:0005886), while in T2 DEGs chloroplast‐associated genes were enriched (Fig. S6A,B). In the MF category, amino acid transmembrane transporter activity (GO:0015171), transferase activity (GO:0016757) and oxidoreductase activity (GO:0016491) genes were highly represented in T1 DEGs, while T2 DEGs were enriched for Chl and pigment binding (GO:0016168, GO:0031409) (Fig. S6C,D). To increase our understanding of the biological functions of the DEGs, pathway enrichment analysis was done by mapping the DEGs to the KEGG database categories. Thirteen KEGG pathways were significantly enriched at T1 (adjusted *P* < 0.005) with secondary metabolic pathways, such as carotenoid biosynthesis (fold enrichment 6.0) and flavonoid biosynthesis (fold enrichment 5.5) particularly indicated (Table 1). Pathway enrichment of T2 DEGs also identified metabolic pathways, including biosynthesis of secondary metabolites as well as photosynthesis‐related pathways (Table 1). In general, secondary metabolic genes were highly represented in T1 DEGs, while Chl‐ and photosynthesis‐related genes were enriched in T2 DEGs. Among the genes upregulated by *AdMYB7* expression in *N*. *benthamiana* was *geranylgeranyl pyrophosphate synthase* (*GGPPS*), which catalyses the formation of geranylgeranyl pyrophosphate, a 20‐carbon substrate for both the carotenoid and the Chl biosynthetic pathways. The carotenoid biosynthesis genes *NbPSY* and *lycopene cyclase* were upregulated by *AdMYB7* at both T1 and T2, while *phytoene desaturase* (*NbPDS*) and *carotenoid isomerase* were differentially expressed at T1, but not T2 (Table 2). Chlorophyll metabolic genes, such as *geranylgeranyl reductase* (*NbGGR*), coding for a key enzyme in the Chl biosynthetic pathway, and *non‐yellowing* 1 (*Stay‐green 1* (*NbSGR1*)), implicated in Chl breakdown, were upregulated by *AdMYB7* at both T1 and T2. We found putative MYB binding motifs in the promoter sequence of *NbSGR1* suggesting a direct activation by MYB7. By contrast, genes such as FAD/NADP oxidoreductase family proteins and the light harvesting Chl*a/b* binding proteins were generally downregulated at T2. The transcriptomic analysis of *AdMYB7* overexpression in *N*.* benthamiana* suggests it may directly regulate the expression of key genes involved in the carotenoid and Chl biosynthetic pathways.

**Table 1:** KEGG pathway enrichment analysis of differentially expressed genes at T1 and T2

Treatment	Pathway term	Number of genes	*P* value	Fold enrichment	Benjamini–Hochberg *P* value
T1	ath01110:Biosynthesis of secondary metabolites	67	8.8E‐08	1.83	0.000
	ath01100:Metabolic pathways	90	3.7E‐05	1.40	0.002
	ath01130:Biosynthesis of antibiotics	29	7.7E‐04	1.92	0.023
	ath01230:Biosynthesis of amino acids	19	2.2E‐03	2.19	0.050
	ath00906:Carotenoid biosynthesis	6	2.6E‐03	6.07	0.046
	ath00061:Fatty acid biosynthesis	6	1.1E‐02	4.40	0.150
	ath00040:Pentose and glucuronate interconversions	8	1.9E‐02	2.90	0.224
	ath04146:Peroxisome	8	2.7E‐02	2.70	0.271
	ath01212:Fatty acid metabolism	7	3.0E‐02	2.93	0.269
	ath00941:Flavonoid biosynthesis	4	3.2E‐02	5.59	0.261
	ath00640:Propanoate metabolism	4	3.7E‐02	5.34	0.268
	ath00400:Phenylalanine, tyrosine and tryptophan biosynthesis	6	4.3E‐02	3.09	0.284
	ath00900:Terpenoid backbone biosynthesis	6	4.5E‐02	3.04	0.280
	ath00945:Stilbenoid, diarylheptanoid and gingerol biosynthesis	6	5.4E‐02	2.89	0.308
	ath00280:Valine, leucine and isoleucine degradation	5	6.4E‐02	3.26	0.336
T2	ath01100:Metabolic pathways	457	1.8E‐17	1.32	0.000
	ath01110:Biosynthesis of secondary metabolites	289	1.7E‐15	1.47	0.000
	ath01130:Biosynthesis of antibiotics	138	8.2E‐12	1.70	0.000
	ath01200:Carbon metabolism	89	7.5E‐10	1.86	0.000
	ath01230:Biosynthesis of amino acids	87	9.5E‐10	1.86	0.000
	ath00195:Photosynthesis	35	1.2E‐07	2.48	0.000
	ath00710:Carbon fixation in photosynthetic organisms	32	2.7E‐07	2.53	0.000
	ath00860:Porphyrin and Chl metabolism	25	5.4E‐07	2.84	0.000
	ath00196:Photosynthesis – antenna proteins	15	3.2E‐06	3.72	0.000
	ath00630:Glyoxylate and dicarboxylate metabolism	30	1.8E‐05	2.21	0.000
	ath00620:Pyruvate metabolism	31	1.0E‐04	2.02	0.001
	ath00260:Glycine, serine and threonine metabolism	26	6.2E‐04	1.97	0.006
	ath00220:Arginine biosynthesis	16	6.4E‐04	2.50	0.006
	ath00010:Glycolysis/gluconeogenesis	36	6.7E‐04	1.74	0.006
	ath00906:Carotenoid biosynthesis	14	8.8E‐04	2.64	0.007
	ath01210:2‐Oxocarboxylic acid metabolism	26	9.7E‐04	1.92	0.007
	ath00250:Alanine, aspartate and glutamate metabolism	19	1.3E‐03	2.16	0.009
	ath00330:Arginine and proline metabolism	20	1.8E‐03	2.06	0.012
	ath00340:Histidine metabolism	10	2.2E‐03	3.03	0.014
	ath00300:Lysine biosynthesis	9	3.9E‐03	3.07	0.024
	ath00290:Valine, leucine and isoleucine biosynthesis	11	4.5E‐03	2.61	0.026
	ath00650:Butanoate metabolism	9	6.2E‐03	2.89	0.034
	ath00061:Fatty acid biosynthesis	15	8.8E‐03	2.05	0.046
	ath04146:Peroxisome	26	1.1E‐02	1.63	0.054
	ath00030:Pentose phosphate pathway	18	1.3E‐02	1.82	0.063
	ath00130:Ubiquinone and other terpenoid‐quinone biosynthesis	13	1.8E‐02	2.03	0.080
	ath01212:Fatty acid metabolism	21	2.3E‐02	1.64	0.098
	ath00280:Valine, leucine and isoleucine degradation	15	2.6E‐02	1.82	0.108
	ath00053:Ascorbate and aldarate metabolism	14	2.7E‐02	1.86	0.108
	ath00020:Citrate cycle (TCA cycle)	19	3.0E‐02	1.65	0.115
	ath00450:Selenocompound metabolism	8	3.3E‐02	2.43	0.123
	ath00640:Propanoate metabolism	9	3.5E‐02	2.23	0.126
	ath00670:One carbon pool by folate	8	5.7E‐02	2.18	0.196
	ath00920:Sulphur metabolism	13	5.8E‐02	1.73	0.193
	ath03010:Ribosome	79	6.0E‐02	1.19	0.195
	ath00561:Glycerolipid metabolism	15	8.0E‐02	1.58	0.245
	ath00400:Phenylalanine, tyrosine and tryptophan biosynthesis	16	8.4E‐02	1.53	0.252
	ath00660:C5‐Branched dibasic acid metabolism	5	9.3E‐02	2.73	0.268
	ath00970:Aminoacyl‐tRNA biosynthesis	28	9.4E‐02	1.33	0.265
	ath00261:Monobactam biosynthesis	6	9.6E‐02	2.34	0.264

**Table 2:** List of differentially expressed genes (DEGs) associated with carotenoid and Chl metabolism in *AdMYB7*‐infiltrated *Nicotiana benthamiana* leaves at T1 and T2

Gene ID	Description	*Arabidopsis* homologue	Function	Log_2_ fold change			
				T1	P	T2	P
NbS00043137g0007.1	Phytoene synthase	AT5G17230.3	Carotenoid biosynthesis	2.35	0.00	0.29	NA
NbS00015854g0001.1	Lycopene cyclase	AT3G10230.2	Carotenoid biosynthesis	1.84	0.00	1.42	0.01
NbS00021970g0004.1	Lycopene cyclase	AT3G10230.1	Carotenoid biosynthesis	1.63	0.01	1.23	0.03
NbS00034641g0019.1	Carotenoid isomerase	AT1G06820.1	Carotenoid biosynthesis	0.79	0.00	−0.56	0.01
NbS00012713g0001.1	Phytoene desaturase	AT4G14210.1	Carotenoid biosynthesis	0.78	0.00	−0.32	0.15
NbS00014185g0010.1	Phytoene desaturase	AT4G14210.1	Carotenoid biosynthesis	0.25	0.22	−0.38	0.00
NbS00037135g0012.1	Zeta‐carotene desaturase	AT3G04870.2	Carotenoid biosynthesis	0.24	0.62	−0.60	0.00
NbS00023329g0002.1	Lycopene cyclase	AT3G10230.1	Carotenoid biosynthesis	0.00	1.00	−0.45	0.01
NbS00060224g0003.1	Lycopene b/e cyclase	AT5G57030.1	Carotenoid biosynthesis	−0.06	1.00	−1.00	0.00
NbS00005253g0012.1	Phytoene synthase	AT5G17230.2	Carotenoid biosynthesis	−0.08	0.99	−0.85	0.00
NbS00022591g0010.1	Zeaxanthin epoxidase	AT5G67030.1	Carotenoid biosynthesis	−0.10	0.96	−0.93	0.00
NbS00020996g0011.1	B‐carotene hydroxylase	AT5G52570.1	Carotenoid biosynthesis	−0.13	0.97	−1.31	0.00
NbS00049932g0002.1	Zeaxanthin epoxidase	AT5G67030.1	Carotenoid biosynthesis	−0.17	0.82	−0.88	0.00
NbS00025533g0004.1	Lycopene b/e cyclase	AT5G57030.1	Carotenoid biosynthesis	−0.19	0.92	−1.02	0.00
NbS00039798g0009.1	Violaxanthin epoxidase	AT2G21860.1	Carotenoid biosynthesis	−0.28	0.67	−0.69	0.00
NbS00008360g0001.1	Geranylgeranyl reductase	AT4G38460.1	Chl metabolism	1.65	0.02	1.72	0.01
NbS00026057g0001.1	Geranylgeranyl reductase	AT4G38460.1	Chl metabolism	0.95	0.22	1.55	0.00
NbS00001236g0040.1	Non‐yellowing 1 (SGR1)	AT4G22920.1	Chl metabolism	1.56	0.01	1.21	0.02
NbS00023456g0015.1	Non‐yellowing 1 (SGR1)	AT4G22920.1	Chl metabolism	0.87	0.22	1.04	0.02
NbS00001353g0003.1	Pheophytinase	AT5G13800.1	Chl metabolism	0.42	0.69	−0.80	0.02
NbS00006039g0020.1	Chlorophyll A‐B binding family protein	AT4G17600.1	Chl metabolism	0.13	0.97	−0.77	0.00
NbS00023607g0024.1	Chlorophyll A‐B binding	AT4G17600.1	Chl metabolism	0.10	0.98	−0.70	0.00
NbS00020769g0006.1	Chlorophyll A‐B binding	AT1G44575.1	Chl metabolism	−0.04	1.00	−0.75	0.00
NbS00035687g0007.1	Chlorophyll A‐B binding	AT1G44575.1	Chl metabolism	−0.04	1.00	−0.93	0.00
NbS00034163g0004.1	FAD/NAD(P)‐binding oxidoreductase protein	AT1G57770.1	Chl metabolism	−0.08	0.99	−0.55	0.01
NbS00036264g0012.1	Pheophytinase	AT5G13800.1	Chl metabolism	−0.28	0.75	−0.64	0.00
NbS00029310g0013.1	Chlorophyll A‐B binding	AT3G22840.1	Chl metabolism	‐0.48	0.81	1.68	0.00

## Discussion

### MYB7 is a transcription factor that regulates the carotenoid pathway

Although the biosynthetic pathways responsible for Chl and carotenoid accumulation have been well described, the mechanisms involved in how they are regulated, such as during fruit development, remains to be fully understood. TFs are one such group of regulators that play crucial roles in many biological and developmental processes in plants by their regulation of spatiotemporal gene expression through recognition of specific DNA sequences in promoters (Mitsuda & Ohme‐Takagi, 2009). The identification of such regulatory genes controlling the accumulation of these pigments in fruit, for instance, provides the knowledge to develop novel cultivars for consumers. In this study, we describe a kiwifruit MYB TF, *MYB7*, that modulates Chl and carotenoid accumulation, via regulation of key metabolic genes. *AdMYB7* was identified as an activator of the *AdLCY‐*β gene promoter, which is a key step in the kiwifruit carotenoid pathway.

The *AdMYB7* regulation of a secondary metabolic pathway is consistent with the function of some of its closely related homologues in other species. However, no homologue has been shown to be involved in the carotenoid biosynthetic pathway. Phylogenetic analysis placed MYB7 in a clade with AtMYB112, AtMYB108 and AtMYB78. AtMYB112 is implicated in the promotion of anthocyanin accumulation under stress treatment by inducing expression of key TFs, such as PRODUCTION OF ANTHOCYANIN PIGMENT 1 (Lotkowska *et al*., 2015) (Fig. 3). MYB108 has been implicated in jasmonate‐mediated stamen maturation in *Arabidopsis* (Mandaokar & Browse, 2009), abscisic acid‐induced cell death (Cui *et al*., 2013) and in pathogen defence in cotton (Cheng *et al*., 2016). Homologues of AtMYB78 have been described as playing a role during response to heat and drought stress in soybean and sorghum (Pereira *et al*., 2011; Johnson *et al*., 2014). In the phylogeny we also included the two recently published MYBs implicated in carotenoid pathway regulation, ElRCP1 and CrMYB68 (Sagawa *et al*., 2016; Zhu *et al*., 2017). These fall into separate clades and do not cluster either together or near *Ad*MYB7. This suggests that there may be a variety of MYBs from unrelated clades involved in carotenoid regulation.

The presence of MYB binding motifs in the *LCY‐*β promoter and the physical binding of *Ad*MYB7 recombinant protein to a promoter fragment is further evidence of *Ad*MYB7's role as a transcriptional regulator in the carotenoid pathway (Fig. 4a). The promoter deletion constructs, which corresponded to the removal of MYB binding motifs, caused reduced promoter activity suggesting the removal of the binding sites affected activation by *Ad*MYB7 (Fig 3b). Finally, it has been reported that the closest homologue to MYB7, AtMYB112, specifically binds to an 8 bp DNA fragment which contains the core sequence (A/T/G)(A/C)CC(A/T)(A/G/T)(A/C)(T/C) (Lotkowska *et al*., 2015). Such a sequence exists in the *AdLCY‐*β gene promoter, upstream of the ATG start site at position −31, AACCATCC. Further analysis is required to characterise this putative binding site, and the other MYB elements in the *AdLCY‐*β promoter, to determine those that are functionally active. For example, more detailed promoter deletions or target site mutagenesis would help confirm the specificity of activation by MYB7 and the other MYBs that trans‐activated *AdLCY‐*β.

### *MYB7* expression modulates Chl and carotenoid accumulation

Expression of the *MYB7* gene in *A. arguta* and *A. macrosperma* fruit, which was high in the early fruit stages and again later during fruit ripening, suggested it may have a functional role during fruit development. The changes in carotenoid and Chl pigment profiles in transiently infiltrated as well as stably transformed *N*. *benthamiana* plants gave an indication that *MYB7* may modulate the accumulation of both pigments, at least in *N*. *benthamiana*. Some of the stably transformed plants displayed a darker green phenotype, which was observed in tissue culture and in soil‐grown plants due to increased accumulation of Chl pigments. However, others with very high transgene expression showed a significant reduction in Chl and carotenoid concentrations, which correlated with changes in gene expression of key genes (Fig. 8), suggesting that transgene expression above a certain threshold may induce a different phenotype. Although this was unexpected, a similar observation was made when expression of an R2R3 MYB, *RCP1*, over a certain threshold in a transgenic *M*. *lewisii* line resulted in a phenotype contrary to the expected overexpression phenotype (Sagawa *et al*., 2016). Furthermore, in Arabidopsis Stay Green (*sgr*) mutants two opposing phenotypes are observed (Li *et al*., 2017). This means that while overexpression of TFs is an effective way to reveal their function, there are certain limitations with such an approach, particularly in a heterologous host. Because TFs may be involved in multiple pathways, their ectopic expression may result in pleiotropic phenotypes or generate a new phenotype (Zhang, 2003). The phenotype of the *AdMYB7* transgenic plants combined with the gene expression observed in fruit suggested *MYB7* may control accumulation of these pigments.

### MYB7 induced expression of key Chl and carotenoid metabolic genes

*AdMYB7* expression in transgenic *N*. *benthamiana* also increased the expression of *NbLCY‐*β as well as other carotenoid pathway genes, such as *NbPSY*,* NbPDS* and *NbZDS*. The upregulation of *NbLCY‐*β in these *AdMYB7* expression lines is consistent with its interaction with the kiwifruit *LCY‐*β promoter and suggests a regulatory role for *MYB7* in the carotenoid pathway is conserved between species. The increased expression of the other pathway genes may indicate a direct activation of their promoters by *Ad*MYB7 or be due to an indirect feedback effect of *NbLCY‐*β expression. It is not uncommon for genes involved in the same metabolic pathway to be coordinately regulated by common TFs (Espley *et al*., 2007). *LCY‐*β catalyses the conversion of *trans*‐lycopene to beta‐carotene and together with *LCY‐*ɛ catalyse the formation of alpha‐carotene. However, this is dependent on the flux controlled by *PSY* and *PDS* genes that encode the first two committed enzymes in the carotenoid pathway. Inhibition of PSY or PDS activity affects carotenoid content and results in a bleached phenotype due to the virtual absence of carotenoid compounds (Fray & Grierson, 1993; McCarthy *et al*., 2004; Qin *et al*., 2007). It has also been shown that overexpression of *LCY‐*β in transgenic carrot lines results in the increased expression of *PSY* genes, suggesting a positive feedback effect controlling the carotenoid pathway (Moreno *et al*., 2013 **)**. Consistent with the increased expression of the carotenoid genes in the transgenic *N*. *benthamiana* plants, the transcriptomic analysis of *AdMYB7*‐infiltrated *N. benthamiana* leaves showed the upregulation of *NbGGPPS*,* NbPSY*,* NbPDS*,* NbCRTISO* and *NbLCY‐*β.

The increased expression of Chl biosynthesis genes in *AdMYB7*‐transgenic *N*. *benthamiana* plants and the upregulation of *NbGGR* and *NbSGR1* in the transcriptome of *AdMYB7*‐infiltrated leaves suggested *MYB7* may play an important role in Chl metabolism. GGR catalyses the reduction of geranylgeranyl pyrophosphate to form phytyl pyrophosphate, which is required to form the phytyl tail in the Chl molecule (Keller *et al*., 1998; Tanaka *et al*., 1999). It has been shown that the phytyl tail affects the properties of the Chl pigment, such as contributing to its conformational stability and is required for binding of the pigment by apoproteins (Fiedor *et al*., 2008). A *Synechocystis* sp. mutant, which lacked phytylation ability, exhibited photooxidative stress and rapid degradation of photosystems under high light conditions (Shpilyov *et al*., 2013). Downregulation of *GGR* transcripts in transgenic tobacco using antisense RNA resulted in plants with less Chl and accumulating geranylgeranylated Chl, an intermediate compound suggesting GGR is required to increase Chl levels in plants (Tanaka *et al*., 1999). The rice yellow green leaf mutant, which has a single base mutation in the rice *GGR* (*OsCHl P*), exhibited reduced Chl levels and arrested chloroplast development (Zhou *et al*., 2013; Wang *et al*., 2014). By contrast, the significant decrease in pigment concentration observed in some of the lines showing high expression of the *AdMYB7* transgene was intriguing. This phenotype suggested MYB7 may cause Chl degradation and/or interfere with plastid development, which is consistent with the upregulation of *NbSGR1* and the consequent downregulation of genes associated with chloroplast and thylakoid membrane organization. *SGR1* promotes Chl degradation by interacting with, and probably recruiting, Chl catabolic enzymes during senescence (Sakuraba *et al*., 2012, 2015; Oda‐Yamamizo *et al*., 2016). SGR proteins have magnesium‐de‐chelatase activity, responsible for the extraction of magnesium from Chl*a* and converting it to pheophytin a, during Chl degradation (Shimoda *et al*., 2016). More recently, an Arabidopsis *sgr1* (*nye1*) and *sgr2* (*nye2*) double mutant was reported to have a stay green senescent leaf and seed phenotype due to Chl accumulation. However, the double mutant produced two distinctive types of seeds: green seeds with accumulation of Chl and seeds that were completely devoid of Chl but contained Chl catabolic intermediates. Because the nongreen seed phenotype increased with prolonged silique exposure to light, it was suggested that the phenotype may be due to a photodynamic effect on the accumulating Chl compounds (Li *et al*., 2017). *Ad*MYB7 activation of *SGR1* to promote Chl degradation would be consistent with the transgenic phenotypes that showed significantly reduced Chl concentrations and would be similar to TFs, such as NAC016 and ANAC046, which activate the promoters of Chl catabolic enzymes (Sakuraba *et al*., 2012; Oda‐Yamamizo *et al*., 2016).

### Involvement of *AdMYB7* in Chl and carotenoid accumulation in fruit

The gene expression pattern of *MYB7* displayed a bimodal pattern with peaks associated with early fruit and fruit ripening stages. This expression pattern together with this TF's ability to modulate Chl and carotenoid accumulation, as well as its induction of key metabolic pathway genes, is consistent with *MYB7* having a role in the transition between unripe and ripe fruit (Guyer *et al*., 2014; Fig. S7). There are examples of common regulators between these two metabolic pathways (Park *et al*., 2007; Luo *et al*., 2013). Phytochrome interaction factor 1 (PIF1) has been implicated in regulating Chl and chloroplast development as well as in carotenogenesis, as a post‐transcriptional regulator of PSY (Toledo‐Ortiz *et al*., 2010; Cheminant *et al*., 2011; Bou‐Torrent *et al*., 2015). *PIF1* is induced by low light conditions created by high Chl levels in the fruit, which subsequently represses *PSY* expression. The removal of this inhibition by reduced Chl content therefore results in increased carotenoid accumulation (Llorente *et al*., 2016).

The data presented here suggest a role for *MYB7* in both Chl and carotenoid metabolism, possibly through transcriptional activation of biosynthetic pathway genes. This further supports recent progress in elucidating a role for MYB TFs in carotenoid regulation. Further analysis of *MYB7* using transgenic kiwifruit with elevated or knockdown expression will increase our understanding of its role.

## Author contributions

C.A‐D., A.C.A. and R.V.E. designed the experiment and reviewed all the data; S.D. and C.A‐D. analysed the promoter transactivation and transgenic plants; A.H.T. provided analysis of the bioinformatic and transcriptome data; C.A‐D. and D.L. performed the HPLC analysis. C.A‐D. wrote the body of the paper and all authors reviewed and edited the manuscript.

## Supporting information

Please note: Wiley Blackwell are not responsible for the content or functionality of any Supporting Information supplied by the authors. Any queries (other than missing material) should be directed to the *New Phytologist* Central Office.

**Fig. S1** Sequence of *Actinidia deliciosa* lycopene beta‐cyclase promoter.
**Fig. S2** Concentrations of Chl and carotenoids in fruit.
**Fig. S3** Gene expression of MYB genes in fruit.
**Fig. S4** Relative expression of carotenoid genes in *A. arguta* and *A. macrosperma* fruit.
**Fig. S5** *Nicotiana benthamiana* leaves infiltrated with *AdMYB7*.
**Fig. S6** GO plot of *MYB7* DEGs.
**Fig. S7** A cartoon of *MYB7* gene expression and pigment accumulation in fruit.
**Table S1** Sequence accession numbers
**Table S2** List of primers used

**Table S3** List of differentially expressed genes induced by agrotransfection with empty vector, compared with mock treatment, at 24 and 72 h post‐infiltration

**Table S4** List of differentially expressed genes induced by MYB7, compared with empty vector treatment, at 24 and 72 h post‐infiltration
