# Supplementary material for: A kiwifruit (Actinidia deliciosa) R2R3‐MYB transcription factor modulates chlorophyll and carotenoid accumulation
Source: New Phytol. 2018 Aug 1;221(1):309–25. doi: 10.1111/nph.15362 (PMC6585760; doi:10.1111/nph.15362)
Supplement: Supplementary file 1 — Fig. S1 Sequence of Actinidia deliciosa lycopene beta‐cyclase promoter. Fig. S2 Concentrations of Chl and carotenoids in fruit. Fig. S3 Gene expression of MYB genes in fruit. Fig. S4 Relative expression of carotenoid genes in A. arguta and A. macrosperma fruit. Fig. S5 Nicotiana benthamiana leaves infiltrated with AdMYB7. Fig. S6 GO plot of MYB7 DEGs. Fig. S7 A cartoon of MYB7 gene expression and pigment accumulation in fruit. Table S1 Sequence accession numbers Table S2 List of primers used [file NPH-221-309-s001.pdf]

***New Phytologist* Supporting Information**

Article title: A kiwifruit (*Actinidia deliciosa*) R2R3-MYB transcription factor modulates chlorophyll and carotenoid accumulation.
Authors: Charles Ampomah-Dwamena, Amali H. Thrimawithana, Supinya Dejnoprat, David Lewis, Richard V Espley, Andrew C Allan
Article acceptance date: 11 June 2018

The following Supporting Information is available for this article:

**Fig. S1** Sequence of *Actinidia deliciosa* lycopene beta-cyclase promoter

**Fig. S2** Concentrations of chlorophyll and carotenoids in fruit

**Fig. S3** Gene expression of MYB genes in fruit

**Fig. S4** Relative expression of carotenoid genes in A. arguta and A. macrosperma fruit

**Fig. S5** *N. benthamiana* leaves infiltrated with *AdMYB7*

**Fig. S6** GOplot of *MYB7* DEGs

**Fig. S7** A cartoon of *MYB7* gene expression and pigment accumulation in fruit

**Table S1** Sequence accession numbers

**Table S2** List of primers used

**Table S3** List of differentially expressed genes induced by agro-transfection with empty vector (EV), compared with mock treatment (Buffer), at 24 and 72 hours post infiltration.

**Table S4** List of differentially expressed genes induced by MYB7, compared with empty vector (EV) treatment, at 24 and 72 hours post infiltration.

>Actinidia **deliciosa LCY-B promoter**

ACTGCTGTAGCCTGTACTGTCAACCTTTCCCTTTTTTTGCTAGGTCGCTAGAAATTCATAATTA
ATGTTACCATTGTAGAAAGTTTTGGCAAGATTTAGGAAGATGTCACCACCAAAATCTTGTTAAA
ATATAAATGATC**TTGATATTA**CAATGAGTGCCCACTGTGGGTTCATGAATTTTAGTAA**CGGGAA**
**TCAAT**CTCTTTAAAATAACGTGGTGGTGAAGTTGTCAACGTGCTACTCATAAATTTGTTGCAAG
CTTAGAGGGTTCATGAATTTTAGCAACCGTAATCGATCTCTTCACAATAACGTGGAGGTGAAGT
GCGAAAGTTTAGTGCACGGGGTGTCCTTTTTTTGGGGGAAAAAAATTGGAATTATTGAAGAAAA
GAAAATGAGGCTACATGTTAGACATCCACGCTCTCTCCTTTGCAACGTAGTGTTTATAGGACCA
CATTTCTTTTTCTCTCGTTAATGGAGAGTGTGTGCTTGGGCCCACATGCCTAACTCA**GAGATTC**
**TAA**AATAGTACTCATTCCAGTACTAAATTGCTTCCCATCAGCACCATGCTTTATTTGAACATAC
TTTAACTACTTGTTGACACGTATAGAATTGGTAAACTAGCATGAATGGACTACTGGAGAGTCCC
TGGTGGGTCCCCAATTCCACCTTGTGACAAATAATTCAAATTGGAGGGAAGACCACTAAGCACT
AAACAAACATTTTGGTCACTGGGCCCACAAACTCCTCTACCCTCGCACACACCAACAAAAAT**AA**
**CCGGTC**AATTTTGTCACTTTATGAATTGTATTAGGACCCACTATGCTACCTCTAGTTATTAACG
ATTTTAT**GGTGAATCTC**GTGCAGCATTCYATGTTGTTTCGTCTTGGCCTGTTTTGAATTTGAGC
CAGAACAGGTAGGAGAGCATCTTTCCCTTGGTACTACTTA**GTGATTCCAC**TTTGTACGTATCT**C**
**CCAAATCTT**TCCTCCATTTATAACCTCTCCTCTCTCTCTCTCTCTCTCCCTCTCTCTCTGAA
GCAACTTCAACCATCCCCACCCATCTGATTTTCTCTCTT**ATG**

**Fig. S1** Sequence of *Actinidia deliciosa* lycopene beta-cyclase promoter fragment with predicted MYB binding sites highlighted. The start codon of *MYB7* is shown at the end of sequence.

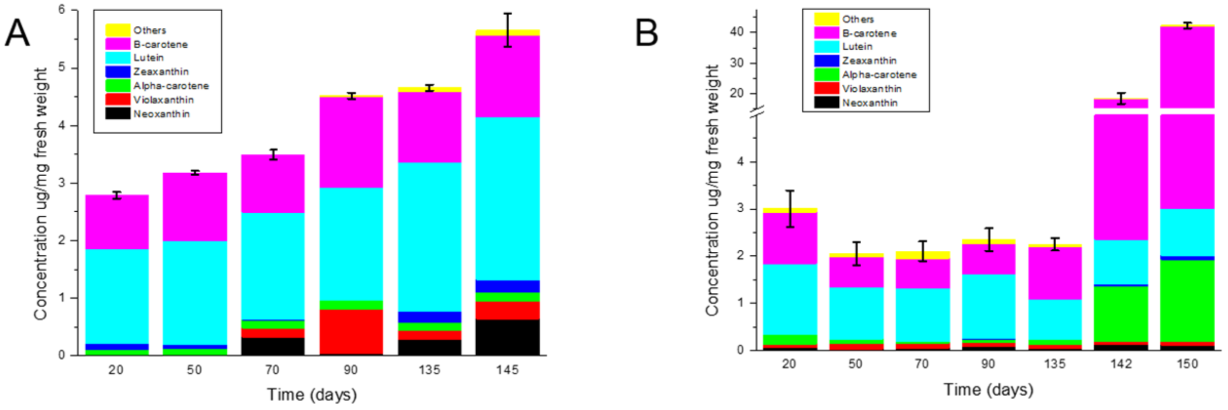


**Fig. S2** Concentration of carotenoid compounds in *Actinidia arguta* (A) and *A. macrosperma* (B) fruit during development. Values are means and standard errors from three biological replicates.

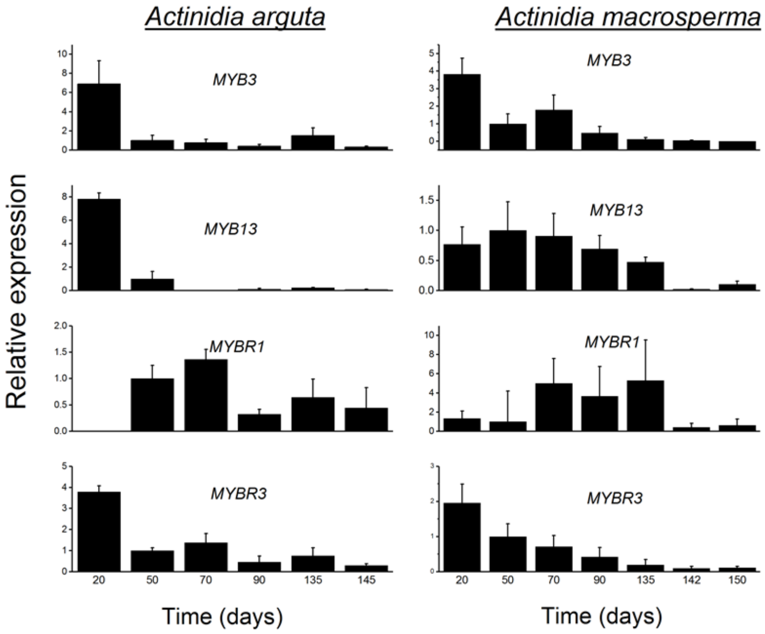


**Fig. S3** Gene expression profile of MYB transcription factor candidates in *Actinidia arguta* and *A. macrosperma* during fruit development. Data were analysed using target-reference ratios as described in Fig. 6 and presented as means and standard error from three biological replicates.

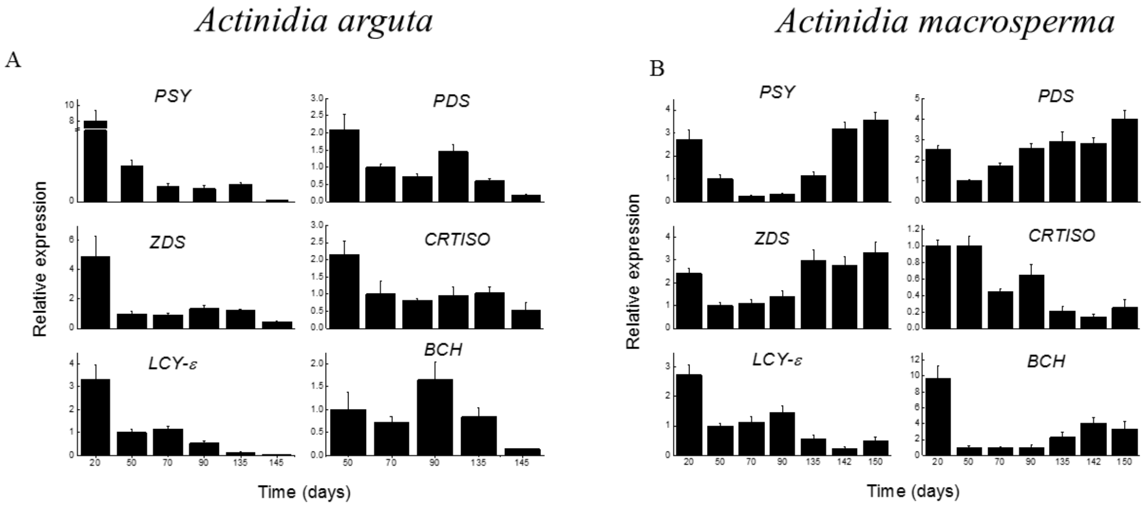

**Fig. S4** Relative expression of carotenoid biosynthetic genes in *A. arguta* (A) and *A. macrosperma* (B) during fruit development. Data were analysed using target-reference ratios (measured with Lightcycler 480 software) using actin as reference gene. Values are means and standard errors from three biological replicates. *PSY*, phytoene synthase; *PDS*, phytoene desaturase; *ZDS*, zeta-carotene desaturase; *CRTISO*, carotene isomerase; *LCY*-ε, lycopene epsilon-cyclase; *BCH*, beta-carotene hydroxylase

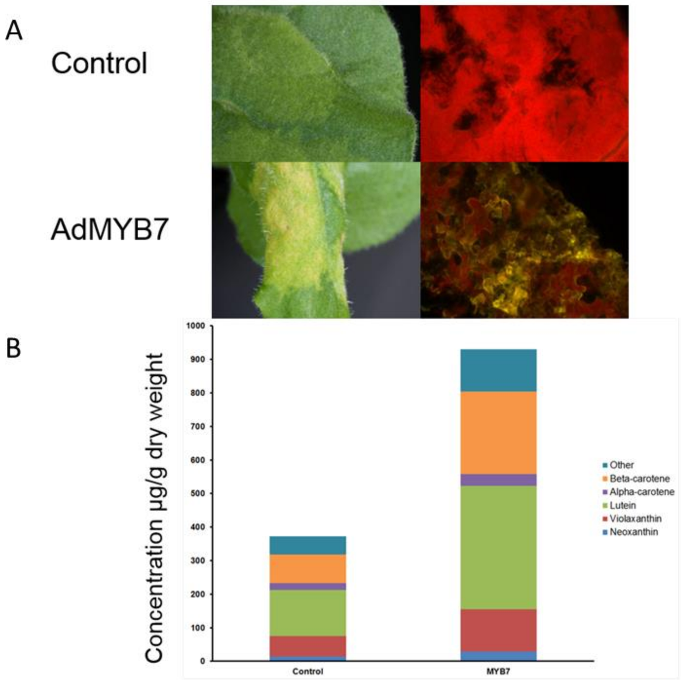


**Fig. S5** *N. benthamiana* leaves infiltrated with *AdMYB7* construct showed an increased carotenoid concentration. A. Leaves infiltrated with *Agrobacterium* constructs of *MYB7* showed yellow colouration when observed under white (left column) or UV luminescence light (right column). B. Pigment concentration in pooled infiltrated leaf patches measured by HPLC. Data are means from pooled replications (n=4).

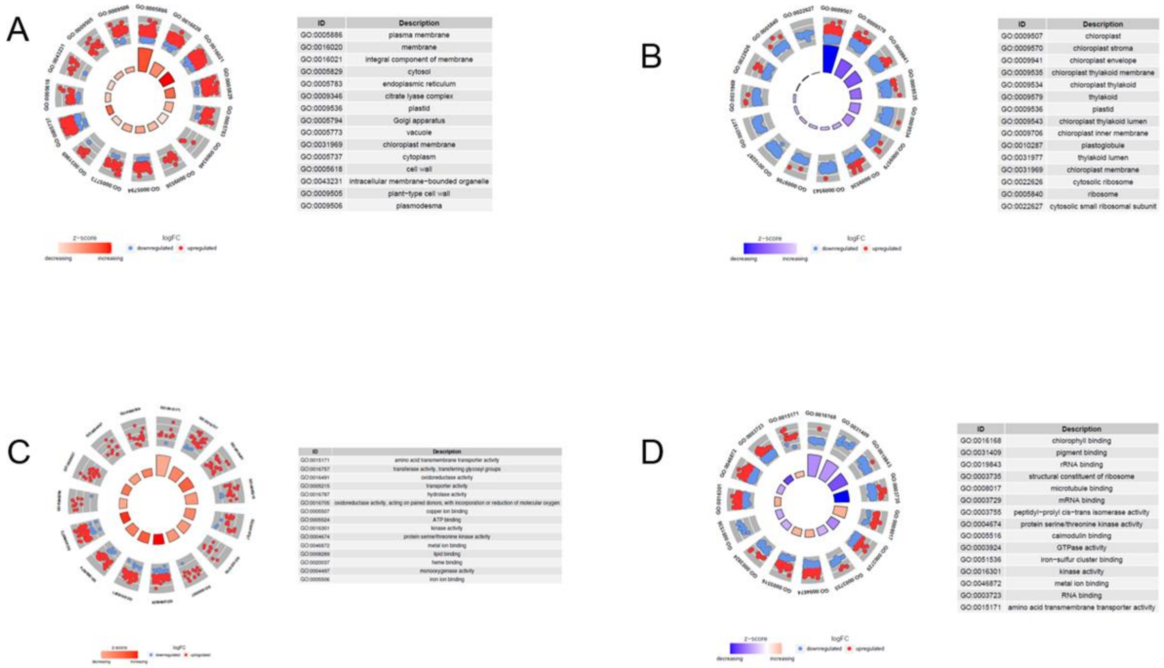


**Fig. S6** GOplot of *MYB7* DEGs in the cellular component (A and B) and molecular function categories (C and D) at T1 and T2, respectively.

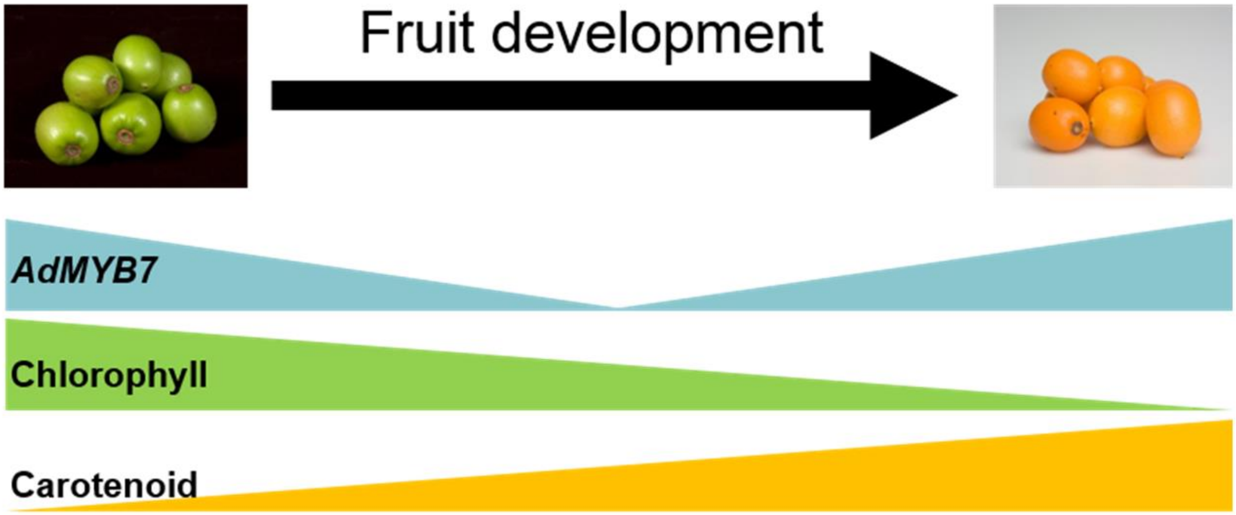


**Fig. S7** A cartoon showing the relationship between *MYB7* gene expression and the accumulation of chlorophyll and carotenoid pigments during fruit development.

**Table S1** Accession numbers and details of amino acid sequences used in the phylogenetic analysis

| Gene name | GenBank accession | Gene ID | Gene name | GenBank accession | Gene ID |
|---|---|---|---|---|---|
| MYB1 | MG581941 | Acc02858 | AtMYB42 | AEE83118.1 | AT4G12350.1 |
| MYB2 | MG581942 | Acc23779 | AtMYB43 | AED92315.1 | AT5G16600.1 |
| MYB3 | MG581943 | Acc23046 | AtMYB44 | AED98326.1 | AT5G67300.1 |
| MYB4 | MG581944 | Acc11236 | AtMYB46 | AED91824.1 | AT5G12870.1 |
| MYB5 | MG581945 | Acc16850 | AtMYB47 | AEE29751.1 | AT1G18710.1 |
| MYB6 | MG581946 | Acc21973 | AtMYB49 | AED96471.1 | AT5G54230.1 |
| MYB7 | MG581947 | Acc24523 | AtMYB4R1 | AEE76047.1 | AT3G18100.1 |
| MYB8 | MG581948 | Acc05669 | AtMYB5 | AEE75369.1 | AT3G13540.1 |
| MYBR1 | MG581949 | Acc30717 | AtMYB50 | AEE33435.1 | AT1G57560.1 |
| MYBR2 | MG581950 | Acc19870 | AtMYB51 | AEE29730.1 | AT1G18570.1 |
| MYBR3 | MG581951 | Acc16728 | AtMYB53 | AED98026.1 | AT5G65230.1 |
| MYBR4 | MG581952 | Acc29824 | AtMYB55 | AEE82062.1 | AT4G01680.2 |
| MYB10 | MG581953 | Acc00493 | AtMYB56 | AED92471.1 | AT5G17800.1 |
| MYB13 | MG581954 | Acc03470 | AtMYB57 | AEE73682.1 | AT3G01530.1 |
| MYB14 | MG581955 | Acc07632 | AtMYB58 | AEE29461.1 | AT1G16490.1 |
| MYB15 | MG581956 | Acc25027 | AtMYB59 | NP_200786.1 | AT5G59780.3 |
| MYB16 | MG581957 | Acc30739 | AtMYB6 | AEE82750.1 | AT4G09460.1 |
| MYB19 | MG581958 | Acc18803 | AtMYB63 | AEE36212.1 | AT1G79180.1 |
| AtMYB1 | AEE74740.1 | AT3G09230.1 | AtMYB64 | AED91629.1 | AT5G11050.1 |
| AtMYB10 | AEE75249.1 | AT3G12820.1 | AtMYB65 | AEE75047.1 | AT3G11440.1 |
| AtMYB100 | AEC07674.1 | AT2G25230.1 | AtMYB66 | AED92073.1 | AT5G14750.1 |
| AtMYB102 | AEE84451.1 | AT4G21440.1 | AtMYB67 | AEE75239.1 | AT3G12720.1 |
| AtMYB103 | AAD40692.1 | AT1G63910.1 | AtMYB68 | AED98108.1 | AT5G65790.1 |
| AtMYB104 | AEC07910.1 | AT2G26950.1 | AtMYB69 | AEE86226.1 | AT4G33450.1 |
| AtMYB107 | AEE73883.1 | AT3G02940.1 | AtMYB7 | AEC06531.1 | AT2G16720.1 |
| AtMYB108 | AEE74402.1 | AT3G06490.1 | AtMYB72 | AEE33352.1 | AT1G56160.1 |
| AtMYB109 | AEE79427.1 | AT3G55730.1 | AtMYB73 | AEE86774.1 | AT4G37260.1 |
| AtMYB11 | AEE80369.1 | AT3G62610.1 | AtMYB74 | AEE82479.1 | AT4G05100.1 |
| AtMYB110 | AEE77522.1 | AT3G29020.2 | AtMYB75 | AEE33419.1 | AT1G56650.1 |
| AtMYB111 | AED95797.1 | AT5G49330.1 | AtMYB76 | AED91195.1 | AT5G07700.1 |
| AtMYB115 | AED94537.1 | AT5G40360.1 | AtMYB77 | CAA74604.1 | AT3G50060.1 |
| AtMYB119 | AED97110.1 | AT5G58850.1 | AtMYB78 | AED95837.1 | AT5G49620.2 |
| AtMYB12 | AEC10843.1 | AT2G47460.1 | AtMYB79 | AEE83284.1 | AT4G13480.1 |
| AtMYB120 | AED96570.1 | AT5G55020.1 | AtMYB8 | Q9SDS8.1 | AT1G35515.1 |
| AtMYB121 | AEE77622.1 | AT3G30210.1 | AtMYB81 | AEC07911.1 | AT2G26960.1 |
| AtMYB122 | AEE35546.1 | AT1G74080.1 | AtMYB82 | AED96239.1 | AT5G52600.1 |
| AtMYB123 | AED93980.1 | AT5G35550.1 | AtMYB83 | AEE74637.1 | AT3G08500.1 |
| AtMYB124 | AEE29151.1 | AT1G14350.1 | AtMYB85 | AEE84639.1 | AT4G22680.1 |

| | | | | | |
|---|---|---|---|---|---|
| AtMYB13 | AEE27955.1 | AT1G06180.1 | AtMYB87 | AEE86837.1 | AT4G37780.1 |
| AtMYB15 | AEE76740.1 | AT3G23250.1 | AtMYB88 | AEC05630.1 | AT2G02820.2 |
| AtMYB16 | AED92146.1 | AT5G15310.1 | AtMYB89 | AED94465.1 | AT5G39700.1 |
| AtMYB17 | AEE80179.1 | AT3G61250.1 | AtMYB9 | AED92335.1 | AT5G16770.1 |
| AtMYB18 | AEE85077.1 | AT4G25560.1 | AtMYB91 | AEC09428.1 | AT2G37630.1 |
| AtMYB19 | AED96193.1 | AT5G52260.1 | AtMYB92 | AED91516.1 | AT5G10280.1 |
| AtMYB20 | AEE34479.1 | AT1G66230.1 | AtMYB94 | AEE78306.1 | AT3G47600.1 |
| AtMYB21 | AEE77366.1 | AT3G27810.1 | AtMYB95 | AEE35591.1 | AT1G74430.1 |
| AtMYB22 | AED94547.1 | AT5G40430.1 | AtMYB96 | AED97611.1 | AT5G62470.2 |
| AtMYB23 | AED94534.1 | AT5G40330.1 | AtMYB97 | AEE85271.1 | AT4G26930.1 |
| AtMYB24 | AED94536.1 | AT5G40350.1 | AtMYB98 | AEE84088.1 | AT4G18770.1 |
| AtMYB25 | AEC09744.1 | AT2G39880.1 | AtMYB99 | AED97596.1 | AT5G62320.1 |
| AtMYB26 | AEE75433.1 | AT3G13890.1 | AtMYB101 | AEC08688.1 | AT2G32460.1 |
| AtMYB27 | AEE79047.1 | AT3G53200.1 | AtMYB105 | AEE34949.1 | AT1G69560.1 |
| AtMYB28 | AED97467.1 | AT5G61420.2 | AtMYB106 | AEE73615.1 | AT3G01140.1 |
| AtMYB29 | AED91194.1 | AT5G07690.1 | AtMYB112 | AEE32236.1 | AT1G48000.1 |
| AtMYB3 | AEE30263.1 | AT1G22640.1 | AtMYB113 | AEE34501.1 | AT1G66370.1 |
| AtMYB30 | AEE77505.1 | AT3G28910.1 | AtMYB114 | AEE34502.1 | AT1G66380.1 |
| AtMYB305 | AEE76887.1 | AT3G24310.1 | AtMYB116 | AEE30608.1 | AT1G25340.1 |
| AtMYB31 | AEE35618.1 | AT1G74650.1 | AtMYB117 | AEE30736.1 | AT1G26780.2 |
| AtMYB32 | AEE86444.1 | AT4G34990.1 | AtMYB14 | AEC08504.1 | AT2G31180.1 |
| AtMYB33 | AED90966.1 | AT5G06100.2 | AtMYB2 | AEC10812.1 | AT2G47190.1 |
| AtMYB34 | AED97393.1 | AT5G60890.1 | AtMYB45 | AEE78473.1 | AT3G48920.1 |
| AtMYB35 | AEE77449.1 | AT3G28470.1 | AtMYB48 | AAY97897 | AT3G46130.1 |
| AtMYB36 | AED96926.1 | AT5G57620.1 | AtMYB52 | AEE29655.1 | AT1G17950.1 |
| AtMYB37 | AED93106.1 | AT5G23000.1 | AtMYB54 | AEE35458.1 | AT1G73410.1 |
| AtMYB38 | AEC09315.1 | AT2G36890.1 | AtMYB60 | AEE28351.1 | AT1G08810.1 |
| AtMYB3R | AF189784.1 | AT3G09370.2 | AtMYB61 | AEE28459.1 | AT1G09540.1 |
| AtMYB3R1 | AEE86112.1 | AT4G32730.2 | AtMYB62 | AEE34781.1 | AT1G68320.1 |
| AtMYB3R2 | AEE81896.1 | AT4G00540.1 | AtMYB70 | AEC07437.1 | AT2G23290.1 |
| AtMYB3R4 | AED91689.1 | AT5G11510.1 | AtMYB84 | AEE78577.1 | AT3G49690.1 |
| AtMYB3R5 | AED90458.1 | AT5G02320.1 | AtMYB86 | AED93580.1 | AT5G26660.1 |
| AtMYB4 | AEE86955.1 | AT4G38620.1 | AtMYB90 | AEE34503.1 | AT1G66390.1 |
| AtMYB40 | AED92019.2 | AT5G14340.1 | AtMYB93 | AEE31733.1 | AT1G34670.1 |
| AtMYB41 | AEE85442.1 | AT4G28110.1 | CrMYB68 | ASK51185 | GI:1236763703 |
| ElRCP1 | ALE33742 | | | | |

**Table S2** Details of primers used in real-time quantitative-PCR

| Name | Gene | Sequence | Species |
|---|---|---|---|
| KFAct F | Actin | TGCATGAGCGATCAAGTTTCAAG | *Actinidia* sp |
| KFAct R | Actin | TGTCCCATGTCTGGTTGATGACT | *Actinidia* sp |
| KFEF1 F | elongation factor 1 alpha | GCACTGTCATTGATGCTCCT | *Actinidia* sp |
| KFEF1 R | elongation factor 1 alpha | CCAGCTTCAAAACCACCAGT | *Actinidia* sp |
| KFLCYB F | lycopene beta-cyclase | GTCGTTCCCGATTCGACGTGAT | *Actinidia* sp |

| | | | |
|---|---|---|---|
| KFLCYB R | lycopene beta-cyclase | TGAAAGTGGCGAGGGATCAACA | *Actinidia* sp |
| KFMYB281 F | MYB7 | GAATCAGAAGCTGCTCTTCGAGGGT | *Actinidia* sp |
| KFMYB281 R | MYB7 | CTCGCTCGTTGTGCAGGATTGAA | *Actinidia* sp |
| KFPSY F | Phytoene synthase | CGAGATTGAAGCCAACGACTAC | *Actinidia* sp |
| KFPSY R | Phytoene synthase | GTTCTCGAAGGGGCAACAATAG | *Actinidia* sp |
| KFPDS F | Phytoene desaturase | AGCAGAAGCCCCCTTCTCAGTG | *Actinidia* sp |
| KFPDS R | Phytoene desaturase | TCCTCTGCAGGTGCAAAAACCA | *Actinidia* sp |
| KFZDS F | Zeta carotene desaturase | TGCATTGTTTGCCACCAAAACAG | *Actinidia* sp |
| KFZDS R | Zeta carotene desaturase | TGCATCCCCACCTGAGATGAA | *Actinidia* sp |
| KFCRTISO F | Carotenoid isomerase | GGACACCAAAGACACACAGGAG | *Actinidia* sp |
| KFCRTISO R | Carotenoid isomerase | GTTGTGTTGAATGGCATCCCTA | *Actinidia* sp |
| KFLCYE F | Lycopene epsilon cyclase | TCGGGTCTACTCTCTCCTCAGC | *Actinidia* sp |
| KFLCYE R | Lycopene epsilon cyclase | GGTCGGAAAGTAGATGCCTGAT | *Actinidia* sp |
| TobLCYB F | lycopene beta-cyclase | GAGCATTGAAGAGGACGAGCAT | *Nicotiana* sp |
| TobPDS F | phytoene destaurase | GCAGGTTTGGGTGGTTTGTCTA | *Nicotiana* sp |
| TobPDS R | phytoene destaurase | TTCCATGCAGCTACCTTTCCAC | *Nicotiana* sp |
| TobPSY F | phytoene synthase | ATTACGTTGCTGGTACGGTTGG | *Nicotiana* sp |
| TobPSY R | phytoene synthase | CGATCCCTAAAGCCAAAGCTG | *Nicotiana* sp |
| TobZDS F | zeta-carotene desaturase | GACCTGATCAGAAGACGCCAGT | *Nicotiana* sp |
| TobZDS R | zeta-carotene desaturase | GCAGAAGCTTGCCTACCTGAAA | *Nicotiana* sp |
| ALAD F | delta-aminolevulinic acid dehydratase | CCTCTCCCTCTCTTCCTCTATTCTG | *Nicotiana* sp |
| ALAD R | delta-aminolevulinic acid dehydratase | CTGTCACCTCAAACTTCACAGCTC | *Nicotiana* sp |
| CHLX F | protochlorophyllide oxidoreductase | GTACACCAGTGTAGTGGGATATGGAG | *Nicotiana* sp |
| CHLX R | protochlorophyllide oxidoreductase | CAGACTACACTTATGCCAGCTTCG | *Nicotiana* sp |
| Mgch F | magnesium chelatase | AAGTACCCGGAGACTGTTGCTCT | *Nicotiana* sp |
| Mgch R | magnesium chelatase | GAGTCTGTAACTGGCCTAACACCA | *Nicotiana* sp |
| MPEc F | magnesium protoporphyrin ester cyclase | CGAGTTGTGGTTCAAACTCTGC | *Nicotiana* sp |
| MPEc R | magnesium protoporphyrin ester cyclase | TCAAGAGGATCCCTCTGATTGC | *Nicotiana* sp |
| PGBD F | porphobilinogen deaminase | GAGGGGGTAGTCCAAGCTACATTA | *Nicotiana* sp |
| PGBD R | porphobilinogen deaminase | AACTGCCGGTAGCATATCCTCT | *Nicotiana* sp |
| Prot F | protoporphyrinogen oxidase | GTCGGGTGCTACTCTTGAACTACA | *Nicotiana* sp |
| Prot R | protoporphyrinogen oxidase | CTGAGGTCACGATCAACTACTTCC | *Nicotiana* sp |
| TobACTIN F | Actin | AAGGTAGTCGCACCACCAGAGAG | *Nicotiana* sp |
| TobACTIN R | Actin | TGACTCGTCGTACTCTGCCTTTG | *Nicotiana* sp |
| TobCRHB F | beta-carotene hydroxylase | TTTGCTCTCTCAGTTGGTGCTG | *Nicotiana* sp |
| TobCRHB R | beta-carotene hydroxylase | GTTTGTGGTGTGACTCGTGCAT | *Nicotiana* sp |
| TobCRHE F | epsilon carotene hydroxylase | TGACTTGGACGTCATACCTGCT | *Nicotiana* sp |
| TobCRHE R | epsilon carotene hydroxylase | GTCTTCATAGGTCGGAGCGTGT | *Nicotiana* sp |
| TobCRTSO F | carotene isomerase | AAGGTAGTCGCACCACCAGAGAG | *Nicotiana* sp |
| TobCRTSO R | carotene isomerase | TGACTCGTCGTACTCTGCCTTTG | *Nicotiana* sp |
| TobLCYB R | lycopene beta-cyclase | GTTGAGGGATGAACCAGACCAG | *Nicotiana* sp |
| TobLCYE F | lycopene epsilon-cyclase | CTTGCTGCGGAGTCTGCTAAAC | *Nicotiana* sp |

| | | | |
|---|---|---|---|
| TobLCYE R | lycopene epsilon-cyclase | GCCCAAGATCTTTGAACTCGTC | *Nicotiana* sp |
